# Causal associations between gut microbiota, circulating inflammatory proteins, and epilepsy: a multivariable Mendelian randomization study

**DOI:** 10.3389/fimmu.2024.1438645

**Published:** 2024-09-09

**Authors:** Han Yang, Wei Liu, Tiantian Gao, Qifan Liu, Mengyuan Zhang, Yixin Liu, Xiaodong Ma, Nan Zhang, Kaili Shi, Minyu Duan, Shuyin Ma, Xiaodong Zhang, Yuxuan Cheng, Huiyang Qu, Mengying Chen, Shuqin Zhan

**Affiliations:** ^1^ Department of Neurology, the Second Affiliated Hospital of Xi’an Jiaotong University, Xi’an, China; ^2^ Department of Pediatric Surgery, the Second Affiliated Hospital of Xi’an Jiaotong University, Xi’an, China; ^3^ Department of Transplant Surgery, the Second Affiliated Hospital of Xi’an Jiaotong University, Xi’an, China

**Keywords:** gut microbiota, epilepsy, inflammatory proteins, Mendelian randomization, microbiota-gut-brain-axis

## Abstract

**Background:**

Previous studies have suggested that gut microbiota (GM) may be involved in the pathogenesis of epilepsy through the microbiota-gut-brain axis (MGBA). However, the causal relationship between GM and different epilepsy subtypes and whether circulating inflammatory proteins act as mediators to participate in epileptogenesis through the MGBA remain unclear. Therefore, it is necessary to identify specific GM associated with epilepsy and its subtypes and explore their underlying inflammatory mechanisms for risk prediction, personalized treatment, and prognostic monitoring of epilepsy.

**Methods:**

We hypothesized the existence of a pathway GM-inflammatory proteins-epilepsy. We found genetic variants strongly associated with GM, circulating inflammatory proteins, epilepsy and its subtypes, including generalized and partial seizures, from large-scale genome-wide association studies (GWAS) summary data and used Multivariate Mendelian Randomization to explore the causal relationship between the three and whether circulating inflammatory proteins play a mediating role in the pathway from GM to epilepsy, with inverse variance weighted (IVW) method as the primary statistical method, supplemented by four methods: MR-Egger, weighted median estimator (WME), Weighted mode and Simple mode.

**Results:**

16 positive and three negative causal associations were found between the genetic liability of GM and epilepsy and its subtypes. There were nine positive and nine negative causal associations between inflammatory proteins and epilepsy and its subtypes. Furthermore, we found that C-X-C motif chemokine 11 (CXCL11) levels mediated the causal association between Genus Family XIII AD3011 group and epilepsy.

**Conclusion:**

Our study highlights the possible causal role of specific GM and specific inflammatory proteins in the development of epilepsy and suggests that circulating inflammatory proteins may mediate epileptogenesis through the MGBA.

## 1 Introduction

Epilepsy, a chronic disorder characterized by the presence of at least one non-provoked epileptic seizure with high recurrence risk, is one of the most common neurological disorders (1), with more than 50 million cases of epilepsy globally, accounting for more than 0.5% of the global economic burden of the disease (2). According to the classification proposed by the International League Against Epilepsy (ILAE) in 2017, epilepsy can be classified into four categories: Generalized Epilepsy (GE), Focal Epilepsy (FE), Combined Generalized and Focal Epilepsy, and Unknown Epilepsy (3). Antiepileptic drugs are the primary and effective means of epilepsy treatment, and there are more than 25 different types of antiepileptic drugs available. However, one-third of epileptic patients still fail to achieve long-term remission with the application of drugs (4). Therefore, it is necessary to explore the pathogenic mechanisms of epilepsy further to provide new therapeutic approaches for the treatment of drug-resistant epilepsy (DRE).

Gut microbiota (GM) is a complex community of bacteria residing in the intestinal tract. There are about 800 species of human GM, mainly composed of the phylum of Firmicutes and Bacteroides (about 94%), of which Clostridia dominate the phylum Firmicutes (about 95%) (5). As an essential regulator of host disease susceptibility, the GM plays a role in basic neurogenic processes such as blood-brain barrier (BBB) formation, myelin sheath formation, neurogenesis, and microglia maturation (6). In recent years, several studies have shown that there is a statistical difference in the fecal microbiota composition between epileptic patients and healthy populations and that modulating the composition of GM in epileptic patients or epilepsy model rats using the ketogenic diet (KD), in turn, improves their epileptic symptoms (7–12). These studies have preliminarily investigated the association between GM and epilepsy, suggesting that GM may regulate neurological functions and behaviors and participate in the pathogenesis of various neurological disorders, including epilepsy, through the microbiota-gut-brain axis (MGBA). However, population-based trials are limited mainly by sample size, and the changes in the abundance of GM associated with epilepsy presented in different trials are inconsistent (13). Also, the effects of and specific mechanisms of various GM taxa on epilepsy have not been clarified.

Inflammatory proteins as essential mediators of inflammatory response, especially pro-inflammatory cytokines in microglia and astrocytes, including interleukin-1β (IL-1β), interleukin-6 (IL-6), tumor necrosis factor-α (TNF-α), and other cytotoxic factors, are closely associated with the occurrence of epilepsy (14). Studies have shown that epileptogenesis is associated with an increased, intense, and persistent inflammatory state in the microenvironment of neuro-tissues (15). However, circulating inflammatory proteins can also enter the central nervous system (CNS) by disrupting the BBB, leading to an inflammatory response that promotes neuronal overexcitation and seizure activity (16). Similarly, circulating inflammatory proteins are closely associated with GM. Recent studies have suggested that GM plays a role in inflammatory regulation by influencing the differentiation of inflammatory cell types, cytokine production, and hematopoiesis. Altering the composition of GM and increasing intestinal permeability can result in the leakage of endotoxins into the circulation, contributing to systemic inflammation (17). Based on the above, we hypothesized that circulating inflammatory proteins may be involved in the pathogenesis of epilepsy through the MGBA.

However, few studies have explored the causal association and mechanism of interaction between specific GM and epilepsy and its subtypes, as well as the potential mediators in the chain of causality between GM and epilepsy. Moreover, the complexity of the human intestinal environment, the numerous factors affecting it, and the difficulty of adequately measuring and controlling confounding factors make it costly, lengthy, and challenging to carry out randomized controlled experiments on large populations. Therefore, we used a novel epidemiological method, Mendelian randomization (MR) analysis, to overcome these limitations.

MR analysis detects and quantifies the causal association between exposure and outcome using genetic variants as instrumental variables (IVs), usually single nucleotide polymorphisms (SNPs) (18). This approach reduces confounding due to environmental factors and avoids bias due to reverse causation (19). Genome-wide association studies (GWAS) have identified thousands of genetic variants associated with various complex diseases, pushing the widespread use of MR to a higher stage (20). Several studies have been performed using MR analysis to explore the association between GM and CNS diseases such as stroke (21), Parkinson’s disease (22), cerebral hemorrhage (23), and Alzheimer’s disease (24). We used multivariate MR analysis on this foundation to investigate the causal relationship between GM and epilepsy, its subtypes, and possible mediating variables in their causal chains.

## 2 Methods

### 2.1 Study design

**Figure 1:**
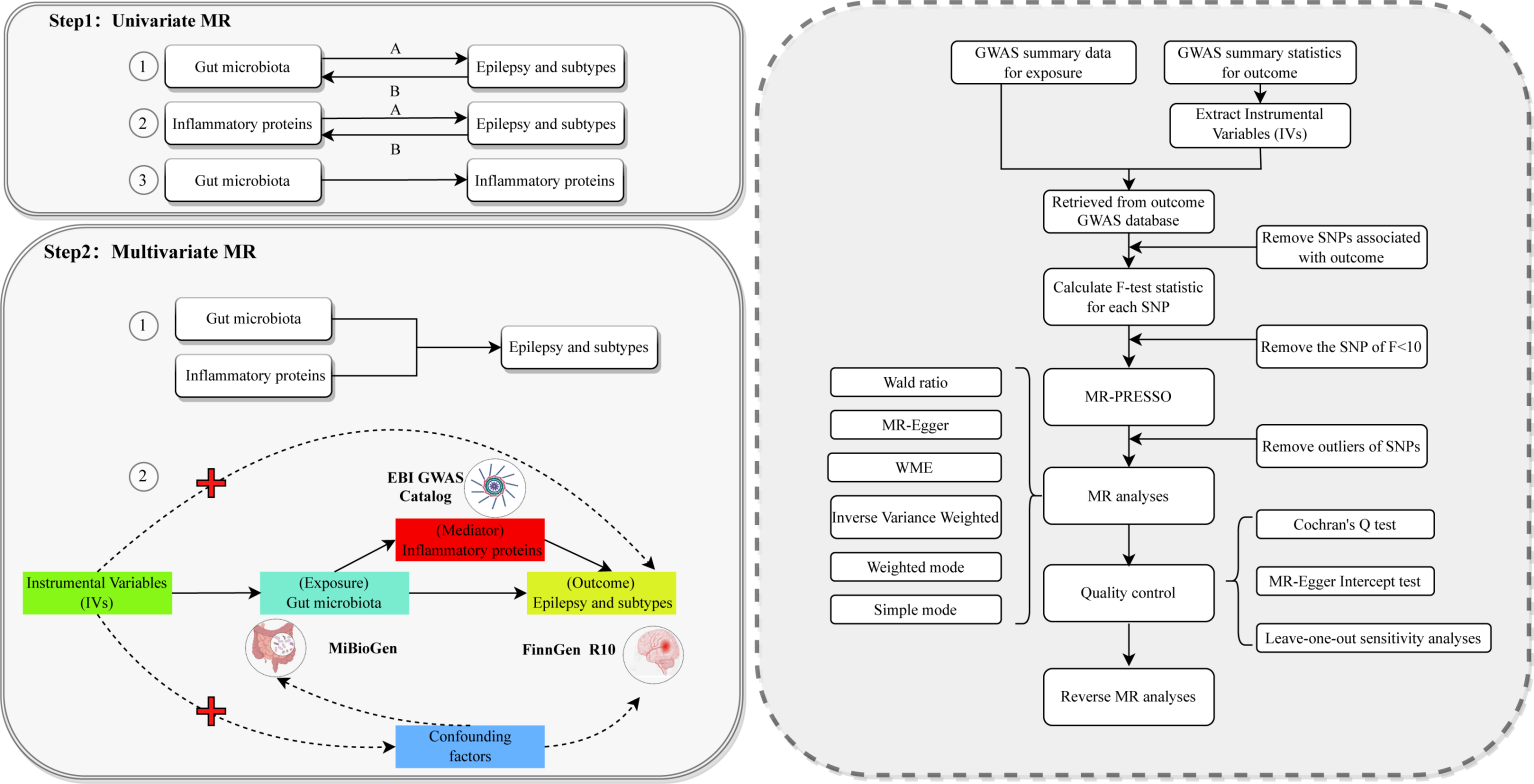
The overall flowchart of this study Step 1-1A represents the causal associations of gut microbiota on epilepsy and subtypes. Step 1-1B represents the reverse causal associations between gut microbiota and epilepsy and its subtypes. Step 1-2A represents the causal associations of inflammatory proteins on epilepsy and subtypes. Step 1-2B represents the reverse causal associations between inflammatory proteins and epilepsy and its subtypes. Step 1-3 represents the causal relationship between gut microbiota significantly associated with epilepsy and its subtypes and inflammatory proteins significantly associated with epilepsy and its subtypes. Each Univariate MR performs the procedure in the dashed box on the right. Step 2-1 represents incorporating positive microbiota and positive proteins obtained from Step 1 into Multivariate MR. Step 2-2 represents the detailed design of this Multivariate MR study of the causal associations of gut microbiota with the risk of epilepsy and its subtypes. MR, Mendelian randomization; GWAS, genome-wide association studies; WME, weighted median estimator; SNP, single nucleotide polymorphism; IVs, instrumental variables.

This study consists of two main components, as shown in **Figure 1**. We first performed univariate Mendelian randomization (UVMR) analyses to assess the causal effects of the GM-inflammatory proteins-epilepsy pathway. We evaluated the causal associations of (i) 211 GM with epilepsy and its subtypes, (ii) 91 inflammatory proteins with epilepsy and its subtypes, and (iii) GM and inflammatory proteins significantly associated with epilepsy. We then analyzed the potential mediating role in the causal relationship between GM and epilepsy by using the significantly associated inflammatory proteins obtained from Step 1, shown in **Figure 1**, as mediating variables and including them in multivariate Mendelian randomization. As in Step 2-2 of **Figure 1**, this design fulfills three basic assumptions of MR, which ensures valid IVs: (i) genetic variants as IVs must be significantly associated with exposure factors; (ii) genetic variants must be independent of any confounding factors affecting exposure and outcome; and (iii) genetic variants can only affect outcome through exposure and not through other pathways (25). This study used the STROBE-MR as the referenced reporting specification (26).

### 2.2 Data sources

The GWAS summary statistics used for statistical analyses are listed in **Table 1**. The human GM GWAS data used in this study was derived from a study by the MiBioGen International Consortium, analyzing genome-wide genotypes and 16S fecal microbiome data from 18,340 participants in 24 cohorts from multiple countries and conducting a large-scale, multi-ancestry, genome-wide meta-analysis of associations between human autosomal genetic variants and GM, with the majority of participants being of European ancestry (16 cohorts, N = 13266). After adjusting for age, sex, technical covariates, and genetic principal components, the results of the study’s microbiome quantitative trait loci (mbQTLs) analyses yielded 211 microbial taxonomically relevant GWAS summary statistics for nine phyla, 16 classes, 20 orders, 35 families, and 131 genera (27).

**Table 1:** Characteristics of the GWAS summary-level data included in this Mendelian randomization analysis.

Trait	Consortium	Samples	Case	Control
Exposure				
211 GM taxa	MiBioGen	18,340	-	-
91 circulating inflammatory proteins	EBI GWAS Catalog	14,824	-	-
Outcome				
Epilepsy	FinnGen (R10)	325,694	12,891	312,803
Generalized epilepsy	FinnGen (R10)	400,506	1,219	399,287
Focal epilepsy	FinnGen (R10)	406,816	7,526	399,290

GM, gut microbiota; GWAS, genome-wide association studies.

The GWAS summary data for circulating inflammatory proteins comes from a recent study that conducted a genome-wide protein quantitative trait locus (pQTL) study of 91 plasma proteins in 14,824 participants from 11 cohorts and combined these data with disease GWAS to characterize the functional effects of disease-associated variants (28).

The GWAS summary data for epilepsy in this study were obtained from the FinnGen Consortium R10 release, which involves 12891 epilepsy patients and 312803 control subjects and is the most recent genome-wide large-scale analysis available. In addition, we also downloaded summary GWAS data for GE and FE (29). FinnGen’s diagnosis of epilepsy is based on G40 in the 10th edition of the International Classification of Diseases (ICD). Under a strict definition, GE and FE are narrower endpoints for epilepsy.

### 2.3 Instrumental variables selection

In this study, P < 5 × 10^-8^ was pre-set as the significance threshold to screen highly correlated SNPs in the GWAS data of GM and inflammatory proteins. Since no SNPs or fewer than 3 SNPs were extracted for specific GM or inflammatory proteins, based on the convention of previously published articles, this study relaxed the threshold requirement by using P < 1 × 10^-5^ and P < 5 × 10^-6^ as the significance thresholds for selecting IVs associated with GM and inflammatory proteins, respectively (24). The selection of all IVs utilized additional criteria to ensure the robustness and accuracy of outcomes: (i) Independent SNPs were clustered according to Linkage disequilibrium (LD) analysis to obtain exposure, with a clustering threshold of R^2^ value < 0.001 and allelic distance > 10000 kb, to ensure that the IVs were independent of each other. If some SNPs were not included in the outcome GWAS, the 1000 Genomes Project’s European sample data was used as the reference standard to find alternative SNPs with R^2^ value > 0.8 under the LD criterion, and if no alternative SNP could be found, this SNP was excluded (30, 31); (ii) Exclusion of palindromic SNPs was performed to prevent alleles from affecting causal outcomes; (iii) Calculation of the F statistic for each IV according to the formula:


$$\text{F}=\frac{\left(\text{N}-\text{K}-1\right)}{\text{K}}\times \frac{{\text{R}}^{2}}{\left(1-{\text{R}}^{2}\right)}$$

where R^2^, N, and K mean the estimated exposure variance explained by IVs, sample size, and the number of IVs, respectively; R^2^ is calculated as


$$R^{2}=2\times EAF\times \left(1-EAF\right)\times \beta^{2}$$

where EAF is the effect allele frequency, β is the allele effect value, and SNPs with F value < 10 were excluded to avoid bias caused by weak IVs (19, 32).

### 2.4 Univariable Mendelian randomization analysis

We first performed three UVMR analyses to assess the causal association of the GM-inflammatory proteins-epilepsy pathway (**Figure 1**). We used the Wald ratio to evaluate causal effects for data in which a single SNP was used as an instrumental variable (32). The Wald ratio, which uses a single IV to estimate the causal effect of exposure (X) on outcome (Y), is the basis for all MR analyses. For data with 2 SNPs as IVs, MR analysis was performed using the inverse variance weighted (IVW) method (33). The IVW method is characterized by regressions that do not take into account the presence of an intercept term and are fitted with the inverse of the variance of the outcome as a weight. Thus, if there is no evidence of horizontal pleiotropy in the IVs, the results of IVW will be determined to be the most reliable. For data with multiple SNPs as instrumental variables, we used IVW, Mendelian randomization of Egger regression (MR-Egger), weighted median estimator (WME), Weighted mode, and Simple mode for MR analysis. The size of the regression intercept of MR-Egger can detect and adjust genetic pleiotropy. The larger the intercept, the greater the possibility of genetic pleiotropy. If the P>0.05 of the pleiotropy test, it is considered that there is no genetic pleiotropy, and the influence of the pleiotropy on the estimation of causal effect can be ignored, but the efficacy of the MR-Egger method is lower than that of the other methods (34). The weighted median method gives unbiased estimates based on the assumption that at least 50% of the IVs are valid (35). Under the assumption that all SNPs are valid instrumental variables, The IVW method provides the most precise effect estimates and is more efficacious than other methods. Therefore, the IVW was used in this study as the primary method to analyze the causal associations. The results of MR were expressed as the odds ratios (ORs) and the corresponding 95% confidence intervals (CIs). We determined the results to be statistically significant when the p-value of IVW was < 0.05 and the IVW was in the same direction as the MR-Egger.

### 2.5 Multivariable Mendelian randomization analysis

We included GM and inflammatory proteins significantly causally associated with epilepsy and its subtypes in mediation analyses and used MVMR methods to validate the potential mediating role of inflammatory proteins in the causal pathway between GM and epilepsy. MVMR is an extension of UVMR that can decompose the total effect between exposure and outcome into direct and indirect effects through mediation. In our study, UVMR was used to estimate the total causal relationship between exposure and outcome, whereas MVMR analyses were used to assess the direct effect of exposure on outcome after adjusting for mediation (36, 37).

### 2.6 Reverse Mendelian randomization analysis

To determine the direction of causal associations and to exclude the influence of reverse causal associations on the results, we performed reverse MR analyses using epilepsy and its subtypes as exposures and significant GM and proteins identified in the forward MR analyses as outcomes, respectively. The conditions for screening IVs and statistical analysis methods were kept consistent with the forward direction to obtain more rigorous results of reverse MR analysis. When the forward MR analysis was statistically significant, but the reverse MR analysis was not, the direction of the causal effect could be further confirmed.

### 2.7 Sensitivity analysis

In order to further test the stability and reliability of the findings and to control the quality of the results, this study conducted heterogeneity tests, horizontal pleiotropy tests, and sensitivity analyses. First, Cochran’s Q test was used to assess the heterogeneity among the effect estimates of IVs, and it was concluded that if the P value was higher than 0.05 and there was no evidence of heterogeneity, the fixed-effects IVW method was the primary method, if the P<0.05, it could be assumed that there was a significant heterogeneity among the effect estimates of IVs, and the random-effects IVW method was used (38). Second, MR-Egger intercept test and Mendelian Randomization Pleiotropy RESidual Sum and Outlier (MR-PRESSO) were used to assess the pleiotropic relationship between IVs and other potential confounders, ensuring that the selected IVs did not affect outcome variables through pathways other than exposure factors. The MR-Egger intercept estimates genetic pleiotropy by analyzing the size of the regression intercept and calculating a p-value, which indicates horizontal pleiotropy in the set of IVs if the p-value is < 0. 05. MR-PRESSO consists of three parts: MR-PRESSO global test, MR-PRESSO outlier test, and MR-PRESSO distortion test. While identifying and eliminating SNPs with horizontal pleiotropic outliers, MR-PRESSO can re-estimate the effect of the IVs set and conduct a difference test between the effect estimates of the IVs set with and without removed outliers to determine whether the outlier SNPs have significantly affected the effect estimates of the original IVs set (39). In addition, we performed sensitivity analyses of the findings using the leave-one-out method, which calculates the results of all remaining IVs by eliminating SNPs from the set of IVs one by one. When there is no statistically significant difference between a particular MR result and the total result, it means that any single SNP cannot drive causality.

Multiple hypothesis tests were conducted in this study. To avoid increasing the probability of one type of error, we used the q-value procedure for False Discovery Rate (FDR) correction to control the false-positive rate of multiple comparisons. In this study, results with FDR q-value < 0.1 were defined as significant; results with P < 0.05 but FDR q-value > 0.1 were defined as nominally significant (40, 41).

All MR analyses were performed using R software (version 4.3.1) under the “TwoSample MR” package (version 0.5.6 https://mrcieu.github.io/TwoSampleMR). The main R packages used were also MR-PRESSO (version 1.0), q-value (version 2.34.0), Forestploter (version 1.1.1), and circlize (version 0.4.15). MR analyses were considered to be statistically significant at P < 0.05.

## 3 Results

### 3.1 Instrumental variable selection

According to the screening criteria for IVs, 195 bacterial traits and 2559 SNPs were obtained in this study, containing 125 in 9 phyla, 224 in 16 classes, 280 in 20 orders, 434 in 31 families, and 1496 in 119 genera, respectively. (Of these, 15 GM were excluded due to unknown information, and 1 GM was excluded due to no eligible SNPs.) The F-statistics for all included variables were greater than 10, indicating no weak instrumental variable bias. (Min = 16.91, Max = 88.42). Detailed information on each GM trait SNP (e.g. effect allele, other allele, beta, standard error, and p-value) is available in **Supplementary Table S1**. We then identified 1820 SNPs associated with 91 inflammatory proteins at a level of P < 5 × 10^-6^, and similarly, the F-statistics of the included variables were all greater than 10. (Min = 20.84, Max = 2740.70) (**Supplementary Table S2**).

### 3.2 UVMR results of GM and inflammatory proteins on epilepsy and epilepsy subtypes

#### 3.2.1 Epilepsy

**Figure 2:**
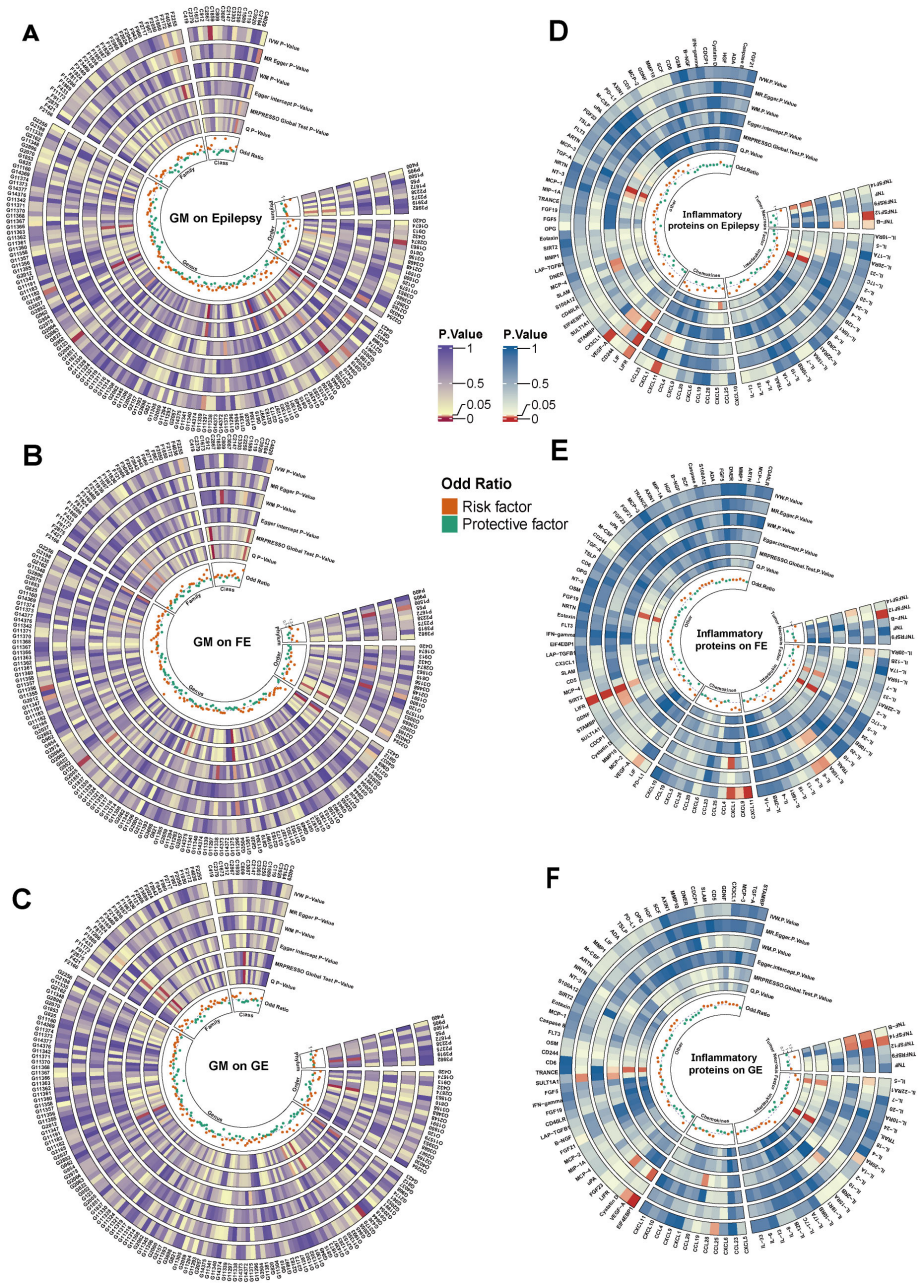
Cyclic heat map of causality associated with epilepsy **(A-C)** represent causal associations of GM with epilepsy and its subtypes, **(D-F)** represent causal associations of inflammatory proteins with epilepsy and its subtypes, and the innermost circle represents the OR value of the IVW method, the outermost abbreviation of **(A-C)** consists of the initial capitalization of taxa names of GM plus the number of GM, and the outermost abbreviations of **(A-F)** are shown in **Supplementary Table S10**. IVW, inverse variance weighted; MR-Egger, Mendelian randomization of Egger regression; WM, weighted median estimator; Q, Cochran’s Q test; GE, Generalized Epilepsy; FE, Focal Epilepsy.

**Figure 3:**
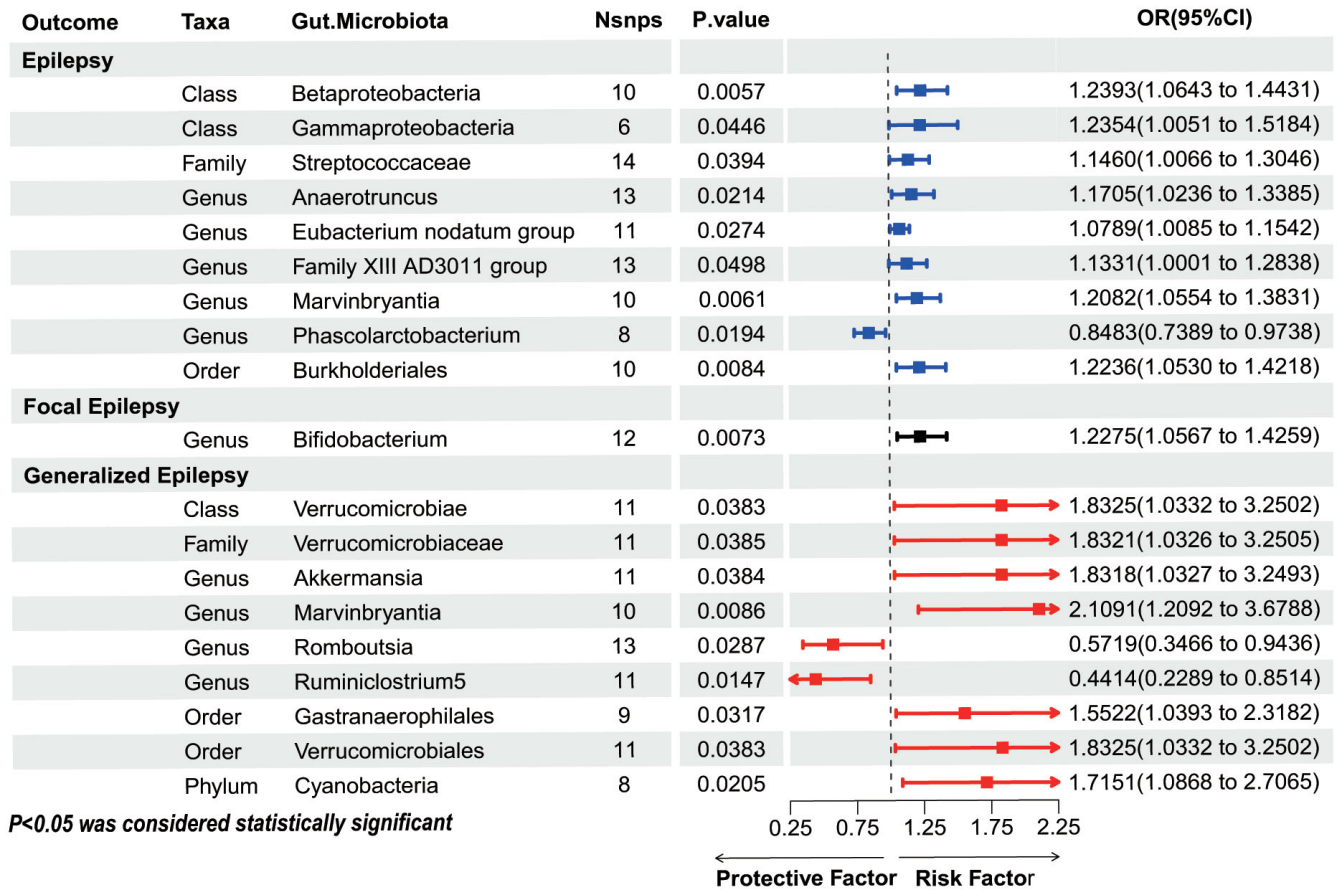
IVW method results on causal associations between GM and epilepsy and its subtypes. Nsnps, number of single nucleotide polymorphisms; OR, odds ratio; CI, confidence intervals.

**Figure 2A** shows the effect of changes in abundance of 192 GM taxa on the risk of epilepsy. The results of our study suggested that a total of eleven GM (including two classes, one order, one family, and seven genera) were associated with epilepsy based on the IVW method, and detailed information on 118 SNPs associated with these eleven GM is shown in **Supplementary Table S3**. The causal associations between GM and epilepsy were also analyzed using MR-Egger, WME, Weighted mode, and Simple mode (**Supplementary Table S4**). Finally, nine epilepsy-associated GM were included, as shown in **Figure 3** (Genus Ruminococcaceae UCG009 was excluded due to the opposite direction of effect value between the IVW method and MR-Egger method, and Genus Bifidobacterium was excluded due to unsatisfied pleiotropy). Class Betaproteobacteria (OR=1.2393, 95%CI =1.0643~ 1.4431, P=0.0057), Class Gammaproteobacteria (OR=1.2354, 95%CI =1.0051~ 1.5184, P=0.0446), Family Streptococcaceae (OR=1.146, 95%CI =1.0066~ 1.3046, P=0.0394), Genus Anaerotruncus (OR=1.1705, 95%CI =1.0236~ 1.3385, P=0.0214), Genus Eubacterium nodatum group (OR=1.0789, 95%CI =1.0085~ 1.1542, P=0.0274), Genus Family XIII AD3011 group (OR=1.1331, 95%CI =1.0001~ 1.2838, P=0.0498), Genus Marvinbryantia (OR=1.2082, 95%CI =1.0554~ 1.3831, P=0.0061), and Order Burkholderiales (OR=1.2236, 95%CI =1.053~ 1.4218, P=0.0084), a total of eight GM were genetically predicted to be associated with an increased risk of epilepsy. Genetic prediction of Genus Phascolarctobacterium (OR=0.8483, 95%CI =0.7389~ 0.9738, P=0.0194) was probably associated with a decreased risk of epilepsy.

**Figure 4:**
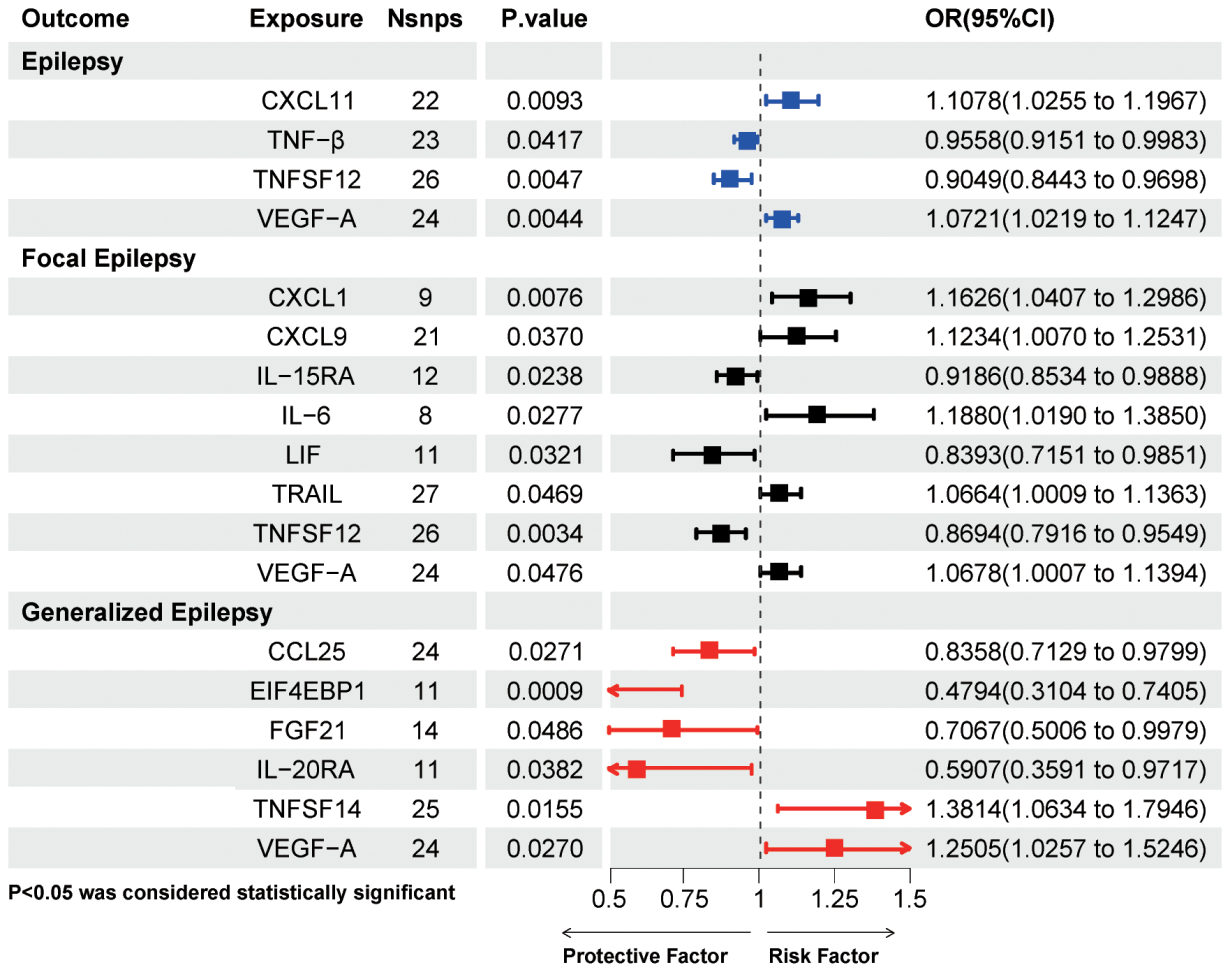
IVW method results on causal associations between circulating inflammatory proteins and epilepsy and its subtypes. Nsnps, number of single nucleotide polymorphisms; OR, odds ratio; CI, confidence intervals; CXCL11, C-X-C motif chemokine 11; TNF-β, Tumor necrosis factor-β; TNFSF12, Tumor necrosis factor ligand superfamily member 12; VEGF-A, Vascular endothelial growth factor A; CXCL1, C-X-C motif chemokine 1; CXCL9, C-X-C motif chemokine 9; IL-15RA, Interleukin-15 receptor subunit alpha; IL-6, interleukin-6; LIF, Leukemia inhibitory factor; TRAIL, TNF-related apoptosis-inducing ligand; CCL25, C-C motif chemokine 25; EIF4EBP1, Eukaryotic translation initiation factor 4E-binding protein 1; FGF21, Fibroblast growth factor 21; IL-20RA, Interleukin- 20 receptor subunit alpha; TNFSF14, Tumor necrosis factor ligand superfamily member 14; VEGF-A, Vascular endothelial growth factor A.

**Figure 2D** presents the effect of changes in 91 circulating inflammatory proteins on the risk of epilepsy. Five inflammatory proteins, including 111 SNPs, were initially screened after the IVW method (**Supplementary Table S5**). The results of Mendelian randomization for the five methods are shown in **Supplementary Table S6**. After a series of sensitivity analyses, Leukemia inhibitory factor receptor (LIFR) levels were excluded due to non-satisfaction of pleiotropy, and thus four inflammatory proteins were causally associated with epilepsy onset at the genetical level (**Figure 4**). Two inflammatory proteins, namely, C-X-C motif chemokine 11(CXCL11) levels (OR=1.1078, 95%CI =1.0255~ 1.1967, P=0.0093) and Vascular endothelial growth factor A (VEGFA) levels (OR=1.0721, 95%CI =1.0219~ 1.1247, P=0.0044) were positively associated with epilepsy. Tumor necrosis factor-β (TNF-β) levels (OR=0.9558, 95%CI =0.9151~ 0.9983, P=0.0417) and Tumor necrosis factor ligand superfamily member 12 (TNFSF12) levels (OR=0.9049, 95%CI =0.8443~ 0.9698, P=0.0047) were negatively associated with epilepsy.

#### 3.2.2 Focal epilepsy

**Figure 2B** presents the effect of abundance variation of 192 GM taxa on FE risk. As shown in **Figure 3**, the genetic prediction of 1 GM, including 12 SNPs, was identified as being associated with FE after IVW screening. Details of the 12 SNPs are shown in **Supplementary Table S3**. The results suggested that Genetic prediction of Genus Phascolarctobacterium per unit increase was associated with a 15.17% reduction in the risk of FE (OR=0.8483, 95%CI =0.7389~ 0.9738, P=0.0194).

**Figure 2E** shows the effect of changes in 91 circulating inflammatory proteins on FE risk. A total of ten inflammatory proteins were associated with FE, and specific information on 176 SNPs is available in **Supplementary Table S5**. After screening by sensitivity test, a total of eight inflammatory proteins were finally included, shown in **Figure 4** (CXCL11 levels was excluded due to the opposite direction of effect value between the IVW method and MR-Egger method, and Leukemia inhibitory factor receptor levels was excluded because it did not satisfy pleiotropy). C-X-C motif chemokine 1 (CXCL1) levels (OR=1.1626, 95%CI =1.0407~ 1.2986, P=0.0076), C-X-C motif chemokine 9 (CXCL9) levels (OR=1.1234, 95%CI =1.007~ 1.2531, P=0.037), IL-6 levels (OR=1.188, 95%CI =1.019~ 1.385, P=0.0277), TNF-related apoptosis-inducing ligand (TRAIL) levels (OR=1.0664, 95%CI =1.0009~ 1.1363, P=0.0469), and VEGFA levels (OR=1.0678, 95%CI =1.0007~ 1.1394, P=0.0476) were associated with an increased risk of FE. While Interleukin-15 receptor subunit alpha (IL15RA) levels (OR=0.9186, 95%CI =0.8534~ 0.9888, P=0.0238), Leukemia inhibitory factor (LIF) levels (OR=0.8393, 95%CI =0.7151~ 0.9851, P=0.0321), and TNFSF12 levels (OR=0.8694, 95%CI =0.7916~ 0.9549, P=0.0034) may decrease the risk of FE.

#### 3.2.3 Generalized epilepsy

**Figure 2C** shows the effect of changes in the abundance of 192 GM taxa on GE risk. The same method was used to explore the causal association between GM and GE, and results showed that 95 SNPs in nine GM were associated with GE (**Supplementary Table S3**). Among them, seven GM, Genus Bifidobacterium (OR=1.2275, 95%CI =1.0567~ 1.4259, P=0.0073), Class Verrucomicrobiae (OR=1.8325, 95%CI =1.0332~ 3.2502, P=0.0383), Family Verrucomicrobiaceae (OR=1.8321, 95%CI =1.0326~ 3.2505, P=0.0385), Genus Akkermansia (OR=1.8318, 95%CI =1.0327~ 3.2493, P=0.0384), Genus Marvinbryantia (OR=2.1091, 95%CI =1.2092~ 3.6788, P=0.0086), Order Gastranaerophilales (OR=1.5522, 95%CI =1.0393~ 2.3182, P=0.0317), Order Verrucomicrobiales (OR=1.8325, 95%CI =1.0332~ 3.2502, P=0.0383) and Phylum Cyanobacteria (OR=1.7151, 95%CI =1.0868~ 2.7065, P=0.0205) may be associated with an increased risk of GE. Two GM, Genus Romboutsia (OR=0.5719, 95%CI =0.3466~ 0.9436, P=0.0287) and Genus Ruminiclostrium5 (OR=0.4414, 95%CI =0.2289~ 0.8514, P=0.0147) were protective factors for GE.

**Figure 2F** presents the effect of changes in 91 circulating inflammatory proteins on the risk of GE. Genetic prediction of 6 inflammatory proteins, a total of 109 SNPs (**Supplementary Table S5**), was associated with the risk of GE. Four inflammatory proteins, namely C-C motif chemokine 25 (CCL25) levels (OR=0.8358, 95%CI =0.7129~ 0.9799, P=0.0271), Eukaryotic translation initiation factor 4E-binding protein 1 (EIF4EBP1) levels (OR=0.4794, 95%CI =0.3104~0.7405, P=0.0009), Fibroblast growth factor 21 (FGF21) levels (OR=0.7067, 95%CI =0.5006~ 0.9979, P=0.0486), and Interleukin- 20 receptor subunit alpha (IL20RA) levels (OR=0.5907, 95%CI =0.3591~ 0.9717, P=0.0382) significantly reduced the risk of GE. Tumor necrosis factor ligand superfamily member 14 (TNFSF14) levels (OR=1.3814, 95%CI =1.0634~ 1.7946, P=0.0155) and VEGFA levels (OR=1.2505, 95%CI =1.0257~ 1.5246, P=0.027) were associated with an increased risk of GE.

### 3.3 Sensitivity analysis

Cochran’s Q test suggested that the results were not statistically significant, indicating that it was not necessary to consider the effect of heterogeneity and genetic pleiotropy on the results, demonstrating the robustness of our findings. Three sets of causal relationships (Genus Bifidobacterium on epilepsy, LIFR on epilepsy, and LIFR on FE) were excluded due to unsatisfied pleiotropy. The regression intercepts of MR-Egger for the remaining data after exclusion did not significantly deviate from 0, with p-values >0.05, indicating no horizontal pleiotropy and suggesting that the relationship between the IVs and the outcome is not affected by potential confounders. In addition, the significance of the MR-PRESSO global test (P > 0.05) revalidated the non-existence of horizontal pleiotropy, and no instrumental variables were identified as potential outliers by the MR-PRESSO outlier test (**Supplementary Table S7**). Leave-one-out analyses showed that after removing SNPs one by one, it turned out that the effect sizes of the including IVs and the total effect sizes were found to be relatively similar, and no SNPs were found yet to have a significant impact on the causal association estimates, see **Additional file 1**. The funnel plot results indicate that when SNPs are used as IVs on a case-by-case basis, the scatter of causal association effects is essentially symmetrically distributed, and there is no potential bias in the results.

### 3.4 Reverse Mendelian randomization analysis

In the reverse MR analysis of GM and epilepsy and its subtypes, the genome-wide significance level was set at P < 1 × 10^-5^, following the principle of consistency with the parameters of the forward MR analysis. Snps were extracted for reverse MR analysis after clustering (R^2^ = 0.001, kb = 10,000) and harmonization. The IVW method (**Supplementary Table S8**) showed no reverse causal association between GM and epilepsy and its subtypes. Also, following the same method and parameter settings as for forward MR (genomic significance level set to P < 5 × 10^-6^, removal of chain disequilibrium parameter set to R^2^ = 0.001, kb = 10,000), there was likewise no reverse causal effect between inflammatory proteins and epilepsy and its subtypes.

### 3.5 MVMR results

The causal effect of epilepsy-associated GM on epilepsy-associated inflammatory proteins is demonstrated in **Supplementary Table S9**, in which seven pairs of potential mediator proteins associated with GM and epilepsy were initially screened. Genus Family XIII AD3011 group was positively associated with CXCL11 levels (OR=1.1398, 95%CI =1.0168~ 1.2776, P=0.0247) among the epilepsy-associated GM and inflammatory proteins. In GM and inflammatory proteins associated with FE, no causal association was found. Among GM and inflammatory proteins associated with GE, a total of five GM, namely, Class Verrucomicrobiae (OR=1.1780, 95%CI =1.0557~ 1.3145, P=0.0034), Family Verrucomicrobiaceae (OR=1.1781, 95%CI =1.0558 ~1.3146, P=0.0034), Genus Akkermansia (OR=1.1782, 95%CI =1.0559~ 1.3146, P=0.0034), Order Gastranaerophilales (OR=1.1215, 95%CI =1.0312~ 1.2196, P=0.0074), and Order Verrucomicrobiales (OR=1.178, 95%CI =1.0557~1.3145, P=0.0034) were positively correlated with CCL25 levels, besides, Phylum Cyanobacteria was positively correlated with FGF21 levels (OR=1.1404, 95%CI =1.0312~ 1.2613, P=0.0106).

We sequentially incorporated seven pairs of GM and inflammatory proteins associated with epilepsy into the mediation analysis using the MVMR method to calculate the indirect effects and proportions mediated by these inflammatory proteins. As shown in **Table 2**, the effect of CXCL11 levels on epilepsy remained significant after adjustment for Genus Family XIII AD3011 group. In contrast, the effect of Genus Family XIII AD3011 group on epilepsy was not significant after adjusting for CXCL11. Therefore, we conclude that CXCL11 levels indirectly affect the association between Genus Family XIII AD3011 group and epilepsy. The mediation percentage was 11.64% (P=0.02126).

**Table 2:** Multivariable MR analyses of the causal associations between gut microbiota, inflammatory proteins, and epilepsy and its subtypes.

Exposure	Mediator	Outcome	Direct effect (β1^*^ ± SE)	P1	Direct effect (β2^*^ ± SE)	P2	Indirect effect (α×β2^*^ ± SE)	Proportion mediated (α×β2^*^/β1)
Genus Family XIII AD3011 group	CXCL11	Epilepsy	0.0997 ± 0.0593	0.0926	0.0887 ± 0.0385	0.0213	0.0116 ± 0.0076	0.1164
Class Verrucomicrobiae	CCL25	GE	0.5645 ± 0.2884	0.2884	-0.0370 ± 0.1208	0.7592	-0.0061 ± 0.0210	-0.0107
Family Verrucomicrobiaceae			0.5644 ± 0.2885	0.0504	-0.0372 ± 0.1208	0.7580	-0.0061 ± 0.0210	-0.0108
Genus Akkermansia			0.5650 ± 0.2883	0.0500	-0.0370 ± 0.1207	0.7595	-0.0061 ± 0.0210	-0.0107
Order Gastranaerophilales			0.2400 ± 0.2300	0.2968	-0.0550 ± 0.1251	0.6604	-0.0063 ± 0.0155	-0.0253
Order Verrucomicrobiales			0.5645 ± 0.2884	0.2884	-0.0370 ± 0.1208	0.7592	-0.0061 ± 0.0210	-0.0107
Phylum Cyanobacteria	FGF21		0.5653 ± 0.2554	0.0269	-0.0961 ± 0.1238	0.4376	-0.0126 ± 0.0182	-0.0223

Beta (β*), standard errors (SE), and P-values (P) are from Multivariate MR shown in Step2 of **Figure 1**. β1* and P1 denote GM’s direct causal effects and P-values on epilepsy and subtypes after adjusting for inflammatory proteins. β2* and P2 denote inflammatory proteins’ direct causal effect and P-values on epilepsy and subtypes after adjusting for GM. α is from Univariate MR shown in Step1-3 of **Figure 1** and indicates the causal effect of GM on inflammatory proteins. β1 is from the Univariate MR shown in Step1-1 of **Figure 1** and represents the total causal effect of GM on epilepsy and subtypes. Indirect effect (α×β2* ± SE) indicates the indirect causal effect of GM on epilepsy and subtypes via inflammatory proteins. Proportion mediated is calculated as the indirect effect divided by the total effect.

MR, Mendelian randomization; GM gut microbiota; CXCL11, C-X-C motif chemokine 11; CCL25, C-C motif chemokine 25; FGF21, Fibroblast growth factor 21.

## 4 Discussion

### 4.1 The value of GM as a biomarker in epilepsy risk prediction, prognostic monitoring, and pharmacodynamics

The GM regulates the CNS through metabolites, neurotransmitters, and inflammatory factors, while the CNS regulates the GM through the sympathetic and parasympathetic branches of the autonomic nervous system or hormonal axes (42). The bi-directional regulatory axis of functional interactions between GM and CNS is known as the MGBA (43, 44). Existing studies have shown that the MGBA plays a vital role in the regulation of a variety of neurological disorders, including Parkinson’s disease, Alzheimer’s disease, depression, and multiple sclerosis (45–48).

Our study comprehensively investigated the causal effects of 211 GM (from phylum to genus) on epilepsy and its subtypes using bidirectional Mendelian randomization, and the results suggested that 3 GM were associated with a reduced risk of epilepsy and its subtypes and 16 GM were associated with an increased risk of epilepsy and its subtypes. These suggest a possible relationship between structural and functional changes in the GM of epileptic patients and their pathogenesis. Existing studies have summarised that GM may be involved in the pathogenesis of epilepsy through a variety of pathways, such as regulating autoimmune and inflammatory responses, synthesizing enteroendocrine signaling and microbial metabolites, influencing the electrical activity of autonomic nerve cells, and improving the activity of the hypothalamic-pituitary-adrenal axis and the endocannabinoid system (13, 42, 49). Research on the mechanisms between epilepsy and GM, as well as the differences in GM between patients with different types of epilepsy and healthy individuals, have made it possible that changes in GM abundance may be a potential biomarker for epilepsy risk prediction, prognostic monitoring, and pharmacodynamics (50).

Our findings suggest that most of the GM associated with an increased risk of epilepsy and its subtypes are from Firmicutes, Proteobacteria, and Verrucomicrobia, with 12 species. A systematic review pooling three human studies and three animal experiments found increases in Firmicutes, Proteobacteria, Verrucomicrobia, and Fusobacteria and decreases in Bacteroidetes and Actinobacteria in epileptic subjects compared to normal controls (51), which is in line with our results, further demonstrating that epilepsy causes dysbiosis of the GM in research subjects. The increased ratio of Firmicutes/Bacteroidetes in patients with epilepsy suggests that this indicator may be potentially valuable for risk prediction in epilepsy, especially DRE.

Our studies have found that different GM has essentially different effects on different epilepsy types. However, we can see that Marvinbryantia is not only a risk factor for epilepsy but also FE, suggesting that Marvinbryantia may be involved in the pathogenesis of different epilepsies through multiple mechanisms. An experiment to evaluate changes in the composition of gut metagenome as well in the fecal metabolomic profile in rats before and after being submitted to status epilepticus (SE)-induced temporal lobe epilepsy (TLE) showed that Desulfovibrio and Marvinbryantia are significantly enriched and positively correlate with excitatory neurotransmitters in microbiota-host metabolism correlation analyses in rats after TLE (52). Research has also shown that the abundance of Marvinbryantia is positively correlated with the levels of inflammatory factors in the serum and intestinal mucosa and negatively correlated with the antioxidant capacity in the serum and the concentration of organic acids in the intestinal contents (53). The results of these studies are similar to ours, and all provide evidence for the pathway by which Marvinbryantia leads to an increased risk of seizures through neuroinflammatory pathways.

Our study showed that three GM, Phascolarctobacterium, Romboutsia, and Ruminiclostrium5, have protective effects against epilepsy and subtypes. An experiment comparing changes in the GM of WAG/Rij(a well-recognized genetic model of absence epilepsy) rats before and after epileptic seizures showed that, At four months of age, the microbiota distribution of WAG/Rij rats was different from that of the control rats, with a significant reorganization of bacterial genera of Firmicutes, of which Clostridiales, Clostridiaceae, and Lachnospiraceae were more abundant and Lactobacillus and Phascolarctobacterium were prominently less abundant (54). This suggested that Phascolarctobacterium may play a partially protective role against the onset of epilepsy in rats during this period. Seizure symptoms became increasingly severe in the WAG/Rij rat group. At the same time, Phascolarctobacterium abundance increased from being significantly lower than that of control rats at one month of age to return to normal control levels by eight months of age, suggesting that GM may be related to pathological aggravation of seizures and demonstrating Phascolarctobacterium may be used as a prognostic biomarker in clinical practice (54). Currently, there are relatively few studies on the effects of Romboutsia and Ruminiclostrium5 on epilepsy, and further studies are still needed to explore their relationship.

Peng et al. (7) found that the abundance of some rare phylum and genera of bacteria, such as Verrucomicrobia and Phascolarctobacterium, was abnormally increased in the DRE group compared with the sensitive group. In a trial comparing the GM composition of infantile spasms patients with and without response to adrenocorticotrophic hormone, the responder group had a decrease in the abundance of Odoribacter, Phascolarctobacterium, and others, as well as an increase in the abundance of Bifidobacterium (55), which provided a basis for the use of Phascolarctobacterium in assessing the pharmacodynamics of epilepsy-related drugs or as a prognostic biomarker. In the future, as research goes further, GM may serve as biomarkers and potential targets for the diagnosis and treatment of epileptic patients, as well as for assessing the efficacy of epilepsy medications.

### 4.2 Involvement of circulating inflammatory proteins in the pathogenesis of epilepsy and its subtypes

Previous studies have shown that regardless of the integrity of the BBB, increased and sustained inflammatory states in the microenvironment of CNS neural tissues have been identified as a significant factor in the physiopathologically induced vicious circle of seizures (56, 57). Circulating inflammatory proteins, critical factors in communicating between the GM and its metabolites and host defense, may also play a role in epilepsy and the induction of a vicious circle through the MGBA (58). Among them, disruption of the BBB and diminished anti-inflammatory effects of short-chain fatty acids (SCFA) may be two main drivers of the vicious cycle of epilepsy (6).

Under a steady-state condition, the GM creates an ultra-low activation of the immune system with stimulation of several types of T cells and macrophages to secrete pro-inflammatory cytokines such as IL-1β and TNF-α. This chronic state of intestinal immune activation can eventually involve the entire body without consequences on health (59, 60). Once the epithelial barrier is damaged by aging, injury, infection, and other factors, it could lead to dysbiosis of the GM and the infiltration of its metabolites, triggering the systemic inflammatory immune response (61). Inflammatory proteins such as TNF-α, IL-6, and IL-1β in serum will increase dramatically in the systemic inflammatory immune state and bind to their special receptors (16), destroying the tight junctions and transendothelial electrical resistance of the BBB (62). At the same time, brain pericytes (a component of the BBB) can also respond to circulating inflammatory signals, such as IL-1β and TNF-α, and transmit these messages to pericytes, aggravating the inflammatory response (63). On the one hand, pericytes may secrete chemokines and regulate leukocyte migration across the brain microvascular endothelial cell barrier by upregulating intercellular adhesion molecule 1 (ICAM-1) and vascular cell adhesion molecule one on endothelial cells (64, 65), on the other hand, pericytes can activate microglia by releasing IL-6 through the Janus kinase-signal transducer and activator of transcriptions (JAK-STAT3) pathway, resulting in breakdown of the BBB and further aggravating the intracranial inflammatory storm, leading to epileptogenesis and a vicious cycle (66).

Some phyla of GM, primarily Bacteroidetes, can ferment insoluble dietary fiber to produce SCFA, including acetate, propionate, and butyrate, which are known to exert anti-inflammatory activity through a variety of mechanisms, thereby reducing the risk of seizures (67). Oxidative stress and neuroinflammation are often co-existing in the brain of patients with epilepsy (68). It has been shown that SCFA, especially butyrate, induces the expression of antioxidant genes, activates cellular antioxidant mechanisms, increases the level of antioxidant enzymes, and thus inhibits the production of pro-inflammatory mediators through histone deacetylase (HDAC) inhibition and nuclear factor erythroid 2-related factor 2 (Nrf2) nuclear translocation (69). SCFA can cross the BBB and activate free fatty acid receptor 2/3 (FFAR2/3) expressed by peripheral immune cells on microglia to exert anti-inflammatory effects, blocking the positive feedback regulation between microglia and pro-inflammatory factors and protecting neurons and glial cells from inflammatory damage (67). In addition, SCFA can directly reduce the expression of pro-inflammatory factors such as IL-6 and TNF-α by disrupting MAPK and NF-κB signaling (70). Once dysbiosis of the GM affects the production of short-chain fatty acids, it may lead to the up-regulation of the inflammatory response and eventually to the development of epilepsy.

Our study used UVMR to explore the causal associations between 91 circulating inflammatory proteins and epilepsy and its subtypes and showed that 18 circulating inflammatory proteins were associated with epilepsy and its subtypes. CXCL11 levels and VEGFA levels increased the risk of epilepsy, CXCL1 levels, CXCL9 levels, IL-6 levels, TRAIL levels, and VEGFA levels were associated with an increased risk of FE and TTNFSF14 levels, VEGFA levels were associated with an increased risk of GE.

We observed that VEGFA promotes increased risk in epilepsy, FE, and GE. In a clinical trial, IL-1β, IL-6, and TNF-α, as well as transforming growth factor-β1 (TGF-β1) and vascular endothelial growth factor (VEGF) mRNA expression were upregulated in the hippocampus after seizures (71, 72). Another clinical study demonstrated that febrile convulsions increase the levels of cytokines IL-1β, IL-6, and TNF-α in cerebrospinal fluid (73). These are similar to our results and further suggest the reliability of subsequent studies in which we investigated the pathway of controlling the inflammatory response to reduce CNS excitability and thus prevent epileptogenesis.

Our results suggest that the effect of CXCL11 levels in increasing the risk of epilepsy remains valuable after adjusting for the effect of GM on epilepsy. CXCL11, also known as interferon inducible T cell α-chemoattractant, belongs to the chemokine CXC subfamily, a member of the non-ELR group, and shares a common C-X-C motif receptor 3 (CXCR3) with CXCL9 and CXCL10 (74). C XCL11 plays a role in immune and inflammatory regulation by recruiting and aggregating immune cells to the site of injury or inflammation through binding to the CXCR3 receptor (75). Previous studies have shown that CXCL11/CXCR3 expression is significantly upregulated in many CNS diseases, such as cerebral ischemic stroke and multiple sclerosis (76, 77). Relatively few studies have examined the relationship between CXCL11/CXCR3 and epilepsy. A study showed that pentylenetetrazole (PTZ)-induced seizure rat models upregulated CXCR3 expression compared to control rats, while pretreatment with sitagliptin reduced CXCL4/CXCR3 expression *in vivo* and *in vitro* (78). These suggest that CXCL11/CXCR3 may be involved in seizure and provide a rationale for targeting the down-regulation of the CXCL11/CXCR3 axis for the treatment of epilepsy.

### 4.3 The prospects of anti-inflammatory effects within MGBA in the treatment of epilepsy

Neuroinflammation itself interacts with epilepsy in a loop (79). Changes in the proportions of specific GM only affect specific cytokine responses (58). Based on this specific GM-Cytokine-disease interaction pattern, treatments targeting modulation of GM to improve neuroinflammation show possible outcomes in epilepsy, especially in DRE patients (79). Currently, new approaches to treating the disease by reversing the ecological dysregulation of GM include KD, Probiotics, and Fecal microbiota transplantation (FMT) (51).

The KD is a pattern of eating a very low-carbohydrate, high-fat diet (80). Several studies have shown that KD can regulate the composition of GM in people with epilepsy and improve epileptic symptoms (8, 9, 11, 12). These beneficial effects may be related to an increased abundance of beneficial bacteria producing short-chain fatty acids, such as Akkermansia muciniphila and Lactobacillus, and a decreased abundance of pro-inflammatory microorganisms, such as Desulfovibrio and Turicibacter (81).

Probiotics are defined as live microorganisms that are beneficial to the host’s health when administered in sufficient amounts. The most common probiotics are Lactobacillus and Bifidobacterium (82). An animal study suggests that a mixture of Lactobacillus rhamnosus, Lactobacillus reuteri, and Bifidobacterium infantis attenuates the severity of PTZ seizures by increasing GABA levels, decreasing nitric oxide production and increasing the antioxidant/oxidant ratio (83). A clinical study that included 70 subjects showed that probiotics had a positive impact on seizure control and improved anxiety, depression and quality of life in TLE patients (84). In an open-label pre-trial, approximately 28.9% of patients with refractory epilepsy had a >50% reduction in seizure frequency after four months of administering a probiotic mixture of 8 bacteria (85). These studies suggest the potential value of altering GM environment through the direct addition of probiotics to control seizure, pending additional trials to validate which bacteria, or mixtures of those bacteria, can maximize the effectiveness of seizure control through altering GM.

FMT is a method of transferring feces from a healthy provider into the gut of a recipient to alter the recipient’s GM directly (86). A FMT from KD-treated mice into control diet mice was sufficient for seizure protection in animal experiments (10). There are case reports that FMT has efficacy in preventing seizure recurrence after withdrawal of antiepileptic drugs (87). In the future, FMT could be used as a promising treatment for epilepsy if purified colony samples highly correlated with elevated epileptic thresholds are available.

In this study, we included GM and inflammatory proteins associated with epilepsy and its subtypes in the mediation analysis and used the MVMR method to explore the causal associations between GM, inflammatory proteins, and epilepsy, and finally obtained the mediating effect of CXCL11 in the causal association between Genus Family XIII AD3011 group and epilepsy. Few studies have examined the relationship between Family XIII AD3011 group and inflammatory proteins. An animal study showed that elevation of the Family XIII AD3011 group at the genus level was strongly associated with hematopoietic toxicity and IL-5 in a mouse model of benzene-induced hematopoietic toxicity (88). In model mice of colitis, Family XIII AD3011 group was positively correlated with the expression level of Aconitate decarboxylase 1 (ACOD1), which is highly expressed in colonic tissues in inflammatory diseases, whose expression is positively correlated with the severity of intestinal inflammation (89). Multiple population trials demonstrated that Family XIII AD3011 group abundance associated with inflammation is elevated in patients with high levels of fatigue, chronic schizophrenia, colitis, and colon cancer (90–92). All of this evidence suggests that the increased abundance of Family XIII AD3011 group causes inflammation, which is generally consistent with our findings. No relevant studies exploring the relationship between Family XIII AD3011 group and epilepsy have been reported. However, our study suggests that CXCL11 and Genus Family XIII AD3011 group may serve as a new target for antiepileptic therapy and also indicates a chain of causality: Genus Family XIII AD3011 leads to epileptogenesis through CXCL11. In the future, animal experiments or large-scale clinical trials can be conducted to investigate the relationship and mechanism of action between the three.

This study is the first large MR study to investigate the mediating role of circulating inflammatory proteins in the causal chain of GM with epilepsy and its subtypes. This study has some limitations: firstly, our study only analyzed European populations. Future studies should consider the use of independent datasets using data from different populations and different research contexts that can help confirm whether the causal relationships we found are generalizable and robust. Secondly, the abundance of GM may also be affected by factors such as diet, gender, medication, and sampling time, and the variance explained by genes may be reduced. Although we explored the role of circulating inflammatory proteins in mediating the relationship between different GM abundances and epilepsy, the mechanisms of how GM affect seizures remain to be investigated, given the complexity of the co-action between GM and inflammatory proteins.

## 5 Conclusions

This study is the first to investigate the causal relationship between GM, circulating inflammatory proteins, and epilepsy and its subtypes. This study showed 16 positive and three negative causal associations between the genetic prediction of GM and epilepsy and its subtypes and nine positive and nine negative correlations between the genetic prediction of inflammatory protein epilepsy and its subtypes. Furthermore, we found that CXCL11 mediates the association between Genus Family XIII AD3011 group and epilepsy. These findings support the role of inflammatory proteins in the pathogenesis of epilepsy through MGBA and provide solid scientific evidence for GM and inflammatory proteins as potential targets for epilepsy in prevention, diagnosis, and treatment. Nonetheless, future studies need to dissect the pathophysiological role of specific GM in epilepsy and its subtypes and further reveal the optimal GM composition to provide a potential approach for the treatment of DRE.

## Data Availability

Publicly available datasets were analyzed in this study. This data can be found here: GWAS summary data for GM genera from the MiBioGen repository was available at https://www.mibiogen.org/. GWAS datasets for 91 circulating inflammatory proteins are available from the EBI GWAS Catalog (https://www.ebi.ac.uk/gwas/downloads/summary-statistics accession numbers GCST90274758 to GCST90274848). GWAS data for epilepsy and its subtypes from the FinnGen consortium were available at https://r10.finngen.fi/.

## Supplementary material

SUPPLEMENTARY TABLE S1 2559 SNPs for the 195 gut microbiota taxa.

SUPPLEMENTARY TABLE S2 1820 SNPs for the 91 circulating inflammatory proteins.

SUPPLEMENTARY TABLE S3 The characteristics of SNPs analyzing the causal effects of gut microbiota on epilepsy and its subtypes.

SUPPLEMENTARY TABLE S4 The causal effects of gut microbiota on epilepsy and its subtypes.

SUPPLEMENTARY TABLE S5 The characteristics of SNPs analyzing the causal effects of circulating inflammatory proteins on epilepsy and its subtypes.

SUPPLEMENTARY TABLE S6 The causal effects of circulating inflammatory proteins on epilepsy and its subtypes.

SUPPLEMENTARY TABLE S7 Mendelian randomization Sensitivity analysis.

SUPPLEMENTARY TABLE S8 Reverse causal effects of epilepsy and its subtypes on the gut microbiota and circulating inflammatory proteins.

SUPPLEMENTARY TABLE S9 Causal effects of gut microbiota associated with epilepsy and its subtypes on circulating inflammatory proteins associated with epilepsy and its subtypes.

SUPPLEMENTARY TABLE S10 Table of abbreviations shown in **Figure 2**.

ADDITIONAL FILE 1 The plots of MR analysis results.

## Glossary

ILAE: International League Against Epilepsy
GE: Generalized Epilepsy
FE: Focal Epilepsy
DRE: drug-resistant epilepsy
GM: gut microbiota
BBB: blood-brain barrier
KD: ketogenic diet
MGBA: microbiota gut-brain axis
IL-1β: interleukin-1β
IL-6: interleukin-6
TNF-α: tumor necrosis factor-α
MR: Mendelian randomization
IVs: instrumental variables
SNPs: single nucleotide polymorphisms
GWAS: genome-wide association studies
UVMR: Univariable Mendelian randomisation
mbQTLs: microbiome quantitative trait loci
pQTL: protein quantitative trait locus
ICD: International Classification of Diseases
LD: Linkage disequilibrium
IVW: inverse variance weighted
MR-Egger: Mendelian randomization of Egger regression
WME: weighted median estimator
ORs: odds ratios
CIs: confidence intervals
MVMR: Multivariable Mendelian randomization
MR-PRESSO: Mendelian Randomization Pleiotropy RESidual Sum and Outlier
FDR: False Discovery Rate
LIFR: Leukemia inhibitory factor receptor
CXCL11: C-X-C motif chemokine 11
VEGFA: Vascular endothelial growth factor A
TNF-β: Tumor necrosis factor-β
TNFSF12: Tumor necrosis factor ligand superfamily member 12
CXCL1: C-X-C motif chemokine 1
CXCL9: C-X-C motif chemokine 9
CXCR3: C-X-C motif receptor 3
TRAIL: TNF-related apoptosis-inducing ligand
IL15RA: Interleukin-15 receptor subunit alpha
LIF: Leukemia inhibitory factor
CCL25: C-C motif chemokine 25
EIF4EBP1: Eukaryotic translation initiation factor 4E-binding protein 1
FGF21: Fibroblast growth factor 21
IL20RA: Interleukin- 20 receptor subunit alpha
TNFSF14: Tumor necrosis factor ligand superfamily member 14
SE: status epilepticus
TLE: temporal lobe epilepsy
WAG/Rij: a well-recognized genetic model of absence epilepsy
CNS: central nervous system
HPA: hypothalamic pituitary adrenal
ICAM-1: intercellular adhesion molecule 1
JAK-STAT: Janus kinase-signal transducer and activator of transcriptions
SCFA: short-chain fatty acids
HDAC: histone deacetylase
Nrf2: nuclear factor erythroid 2-related factor 2
FFAR2/3: free fatty acid receptor 2/3
MAPK: mitogen-activated protein kinases
NF-κB: nuclear factor kappa B
TGF-β1: transforming growth factor-β1
VEGF: vascular endothelial growth factor
PTZ: pentylenetetrazole
FMT: Fecal microbiota transplantation
ACOD1: Aconitate decarboxylase 1
