# Supplementary material for: Causal associations between gut microbiota, circulating inflammatory proteins, and epilepsy: a multivariable Mendelian randomization study
Source: Front Immunol. 2024 Sep 9;15:1438645. doi: 10.3389/fimmu.2024.1438645 (PMC11416947; doi:10.3389/fimmu.2024.1438645)
Supplement: ADDITIONAL FILE 1 — The plots of MR analysis results. [file DataSheet1.docx]

**Additional Figures**

**Figure S1.** MR leave-one-out sensitivity analysis for Gut microbiota on Epilepsy.

**Figure S2.** MR leave-one-out sensitivity analysis for Gut microbiota on Focal Epilepsy.

**Figure S3.** MR leave-one-out sensitivity analysis for Gut microbiota on Generalized Epilepsy.

**Figure S4.** Scatter plots for the effect of Gut microbiota on Epilepsy.

**Figure S5.** Scatter plots for the effect of Gut microbiota on Focal Epilepsy.

**Figure S6.** Scatter plots for the effect of Gut microbiota on Generalized Epilepsy.

**Figure S7.** Forest plots for the effect of Gut microbiota on Epilepsy.

**Figure S8.** Forest plots for the effect of Gut microbiota on Focal Epilepsy.

**Figure S9.** Forest plots for the effect of Gut microbiota on Generalized Epilepsy.

**Figure S10.** Funnel plots for the effect of Gut microbiota on Epilepsy.

**Figure S11.** Funnel plots for the effect of Gut microbiota on Focal Epilepsy.

**Figure S12.** Funnel plots for the effect of Gut microbiota on Generalized Epilepsy.

**Figure S13.** MR leave-one-out sensitivity analysis for circulating inflammatory proteins on Epilepsy.

**Figure S14.** MR leave-one-out sensitivity analysis for circulating inflammatory proteins on Focal Epilepsy.

**Figure S15.** MR leave-one-out sensitivity analysis for circulating inflammatory proteins on Generalized Epilepsy.

**Figure S16.** Scatter plots for the effect of circulating inflammatory proteins on Epilepsy.

**Figure S17.** Scatter plots for the effect of circulating inflammatory proteins on Focal Epilepsy.

**Figure S18.** Scatter plots for the effect of circulating inflammatory proteins on Generalized Epilepsy.

**Figure S19.** Forest plots for the effect of circulating inflammatory proteins on Epilepsy.

**Figure S20.** Forest plots for the effect of circulating inflammatory proteins on Focal Epilepsy.

**Figure S21.** Forest plots for the effect of circulating inflammatory proteins on Generalized Epilepsy.

**Figure S22.** Funnel plots for the effect of circulating inflammatory proteins on Epilepsy.

**Figure S23.** Funnel plots for the effect of circulating inflammatory proteins on Focal Epilepsy.

**Figure S24.** Funnel plots for the effect of circulating inflammatory proteins on Generalized Epilepsy.

**Figure S1.** MR leave-one-out sensitivity analysis for Gut microbiota on Epilepsy.
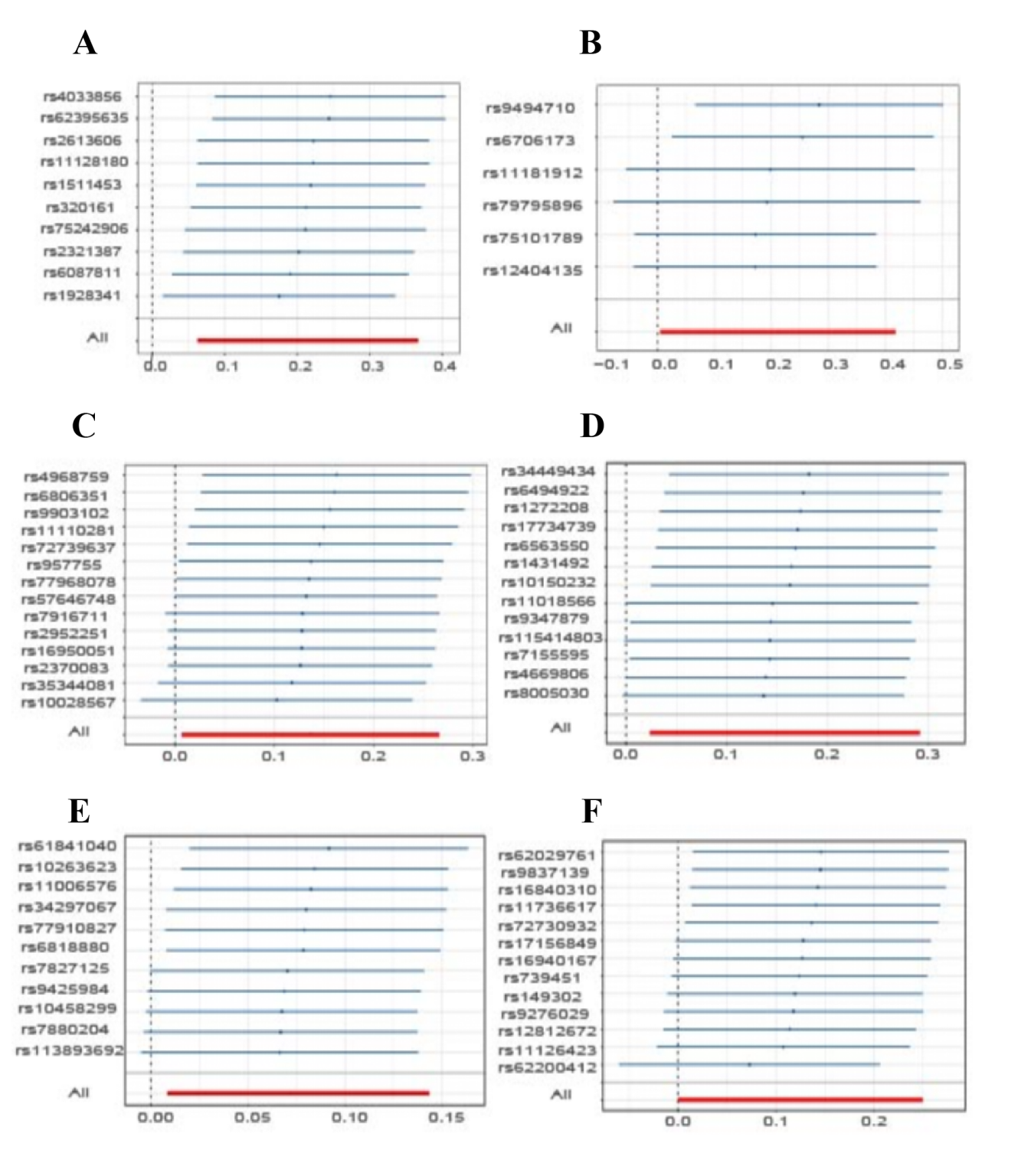


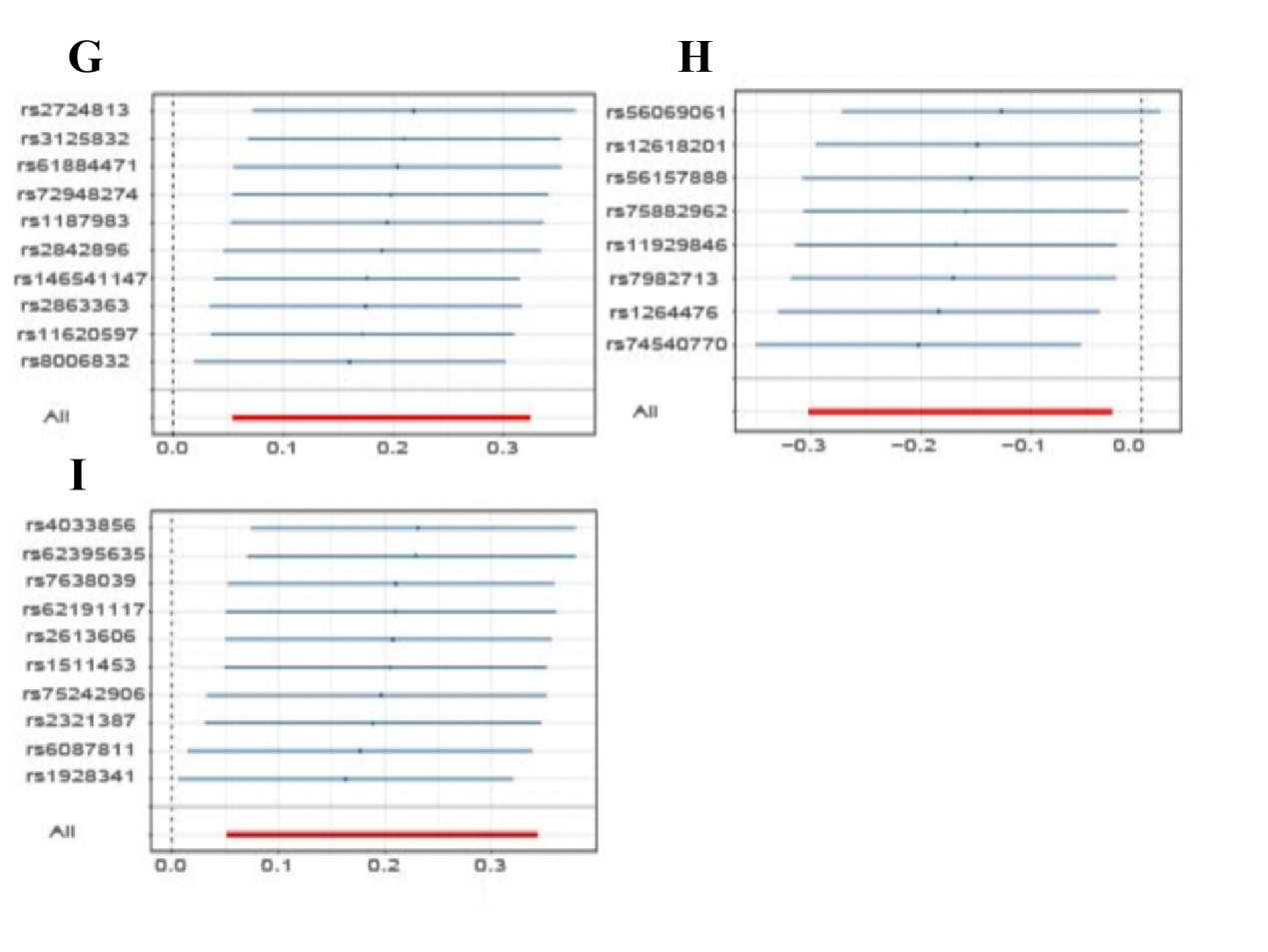


1. Analysis for " Betaproteobacteria " on " Epilepsy "

(B) Analysis for " Gammaproteobacteria" on " Epilepsy "

(C) Analysis for " Streptococcaceae " on " Epilepsy "

(D) Analysis for " Anaerotruncus " on " Epilepsy "

(E) Analysis for " Eubacterium nodatum group " on " Epilepsy "

(F) Analysis for " Family XIII AD3011 group " on " Epilepsy "

(G) Analysis for " Marvinbryantia " on " Epilepsy "

(H) Analysis for " Phascolarctobacterium " on " Epilepsy "

(I) Analysis for " Burkholderiales " on " Epilepsy "

**Figure S2.** MR leave-one-out sensitivity analysis for Gut microbiota on Focal Epilepsy.


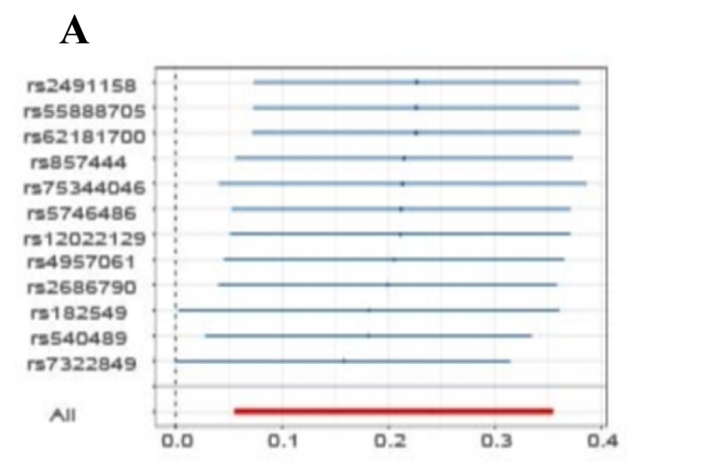


(A) Analysis for " Bifidobacterium " on " Focal Epilepsy "

**Figure S3.** MR leave-one-out sensitivity analysis for Gut microbiota on Generalized Epilepsy.


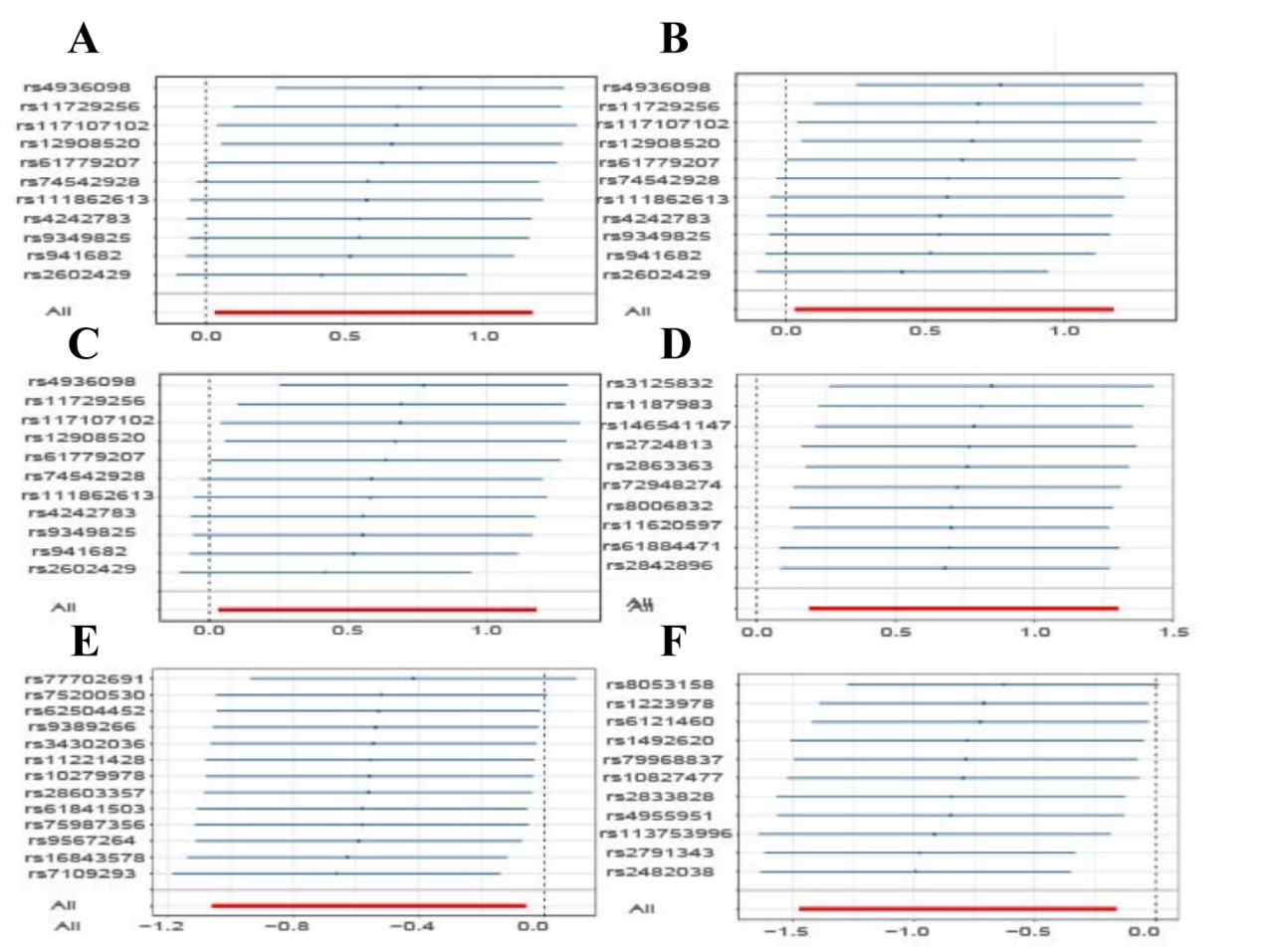


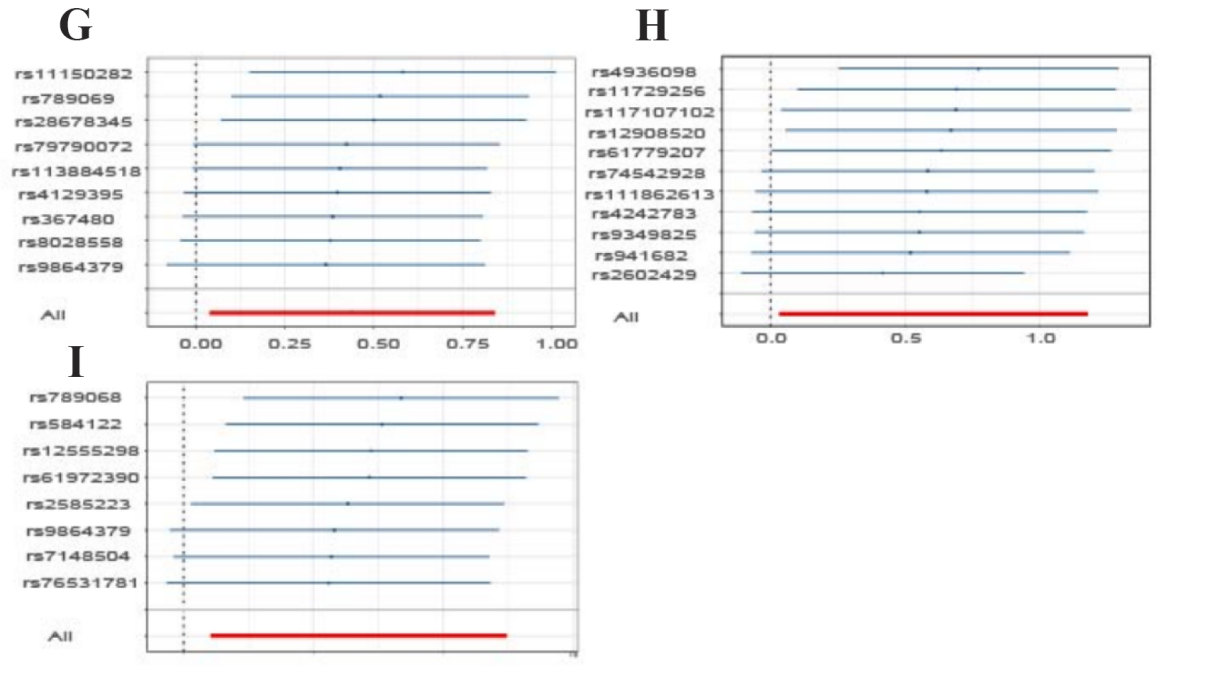


(A) Analysis for " Verrucomicrobiae " on " Generalized Epilepsy"

(B) Analysis for " Verrucomicrobiaceae " on " Generalized Epilepsy "

(C) Analysis for " Akkermansia " on " Generalized Epilepsy "

(D) Analysis for " Marvinbryantia " on " Generalized Epilepsy "

(E) Analysis for " Romboutsia " on " Generalized Epilepsy"

(F) Analysis for " Ruminiclostrium5 " on " Generalized Epilepsy "

(G) Analysis for " Gastranaerophilales " on " Generalized Epilepsy"

(H) Analysis for " Verrucomicrobiales " on " Generalized Epilepsy"

(I) Analysis for " Cyanobacteria " on " Generalized Epilepsy"

**Figure S4.** Scatter plots for the effect of Gut microbiota on Epilepsy.


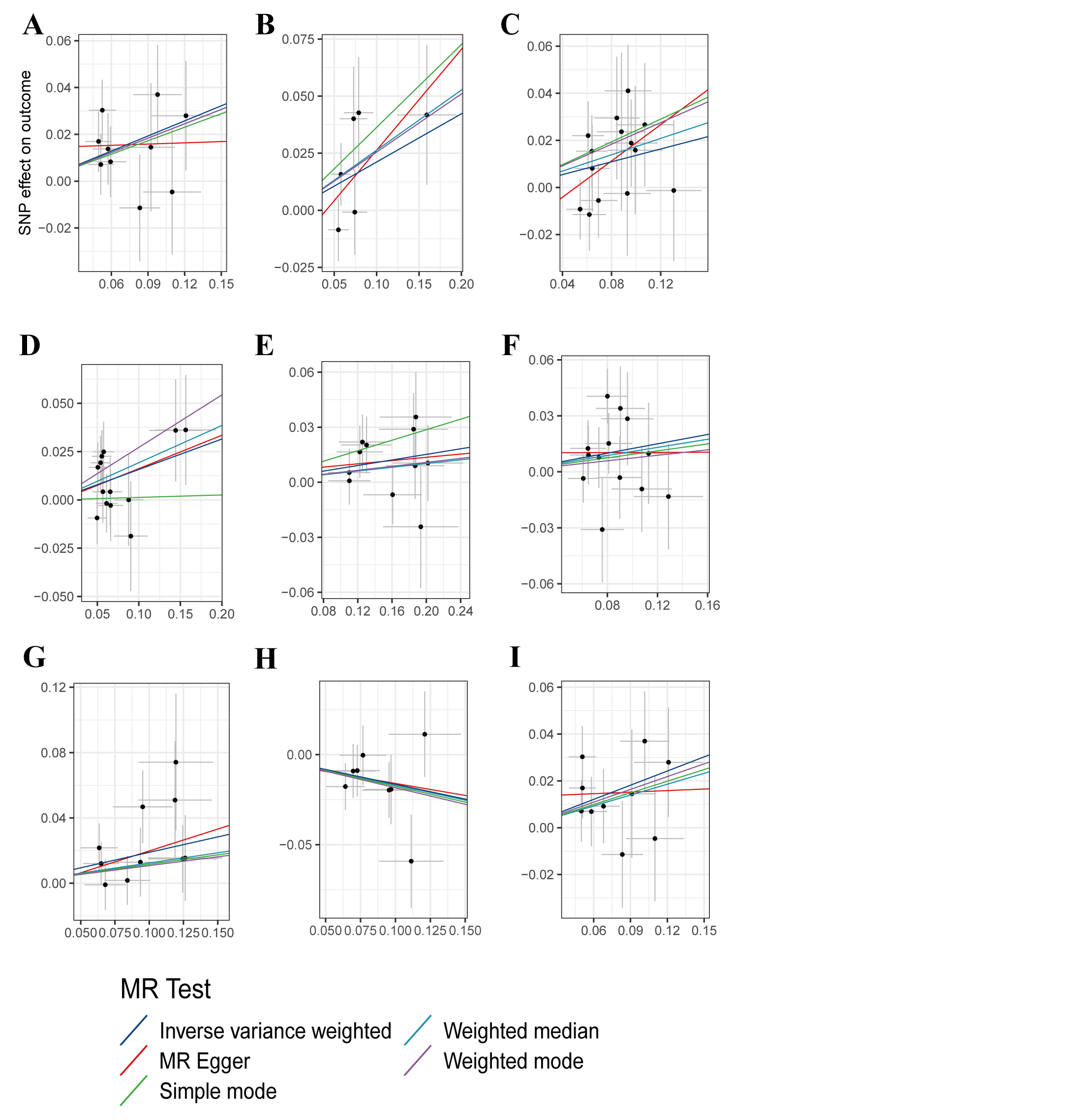


(A)Analysis for " Betaproteobacteria " on " Epilepsy "

(B) Analysis for " Gammaproteobacteria" on " Epilepsy "

(C) Analysis for " Streptococcaceae " on " Epilepsy "

(D) Analysis for " Anaerotruncus " on " Epilepsy "

(E) Analysis for " Eubacterium nodatum group " on " Epilepsy "

(F) Analysis for " Family XIII AD3011 group " on " Epilepsy "

(G) Analysis for " Marvinbryantia " on " Epilepsy "

(H) Analysis for " Phascolarctobacterium " on " Epilepsy "

(I) Analysis for " Burkholderiales " on " Epilepsy "

**Figure S5.** Scatter plots for the effect of Gut microbiota on Focal Epilepsy.


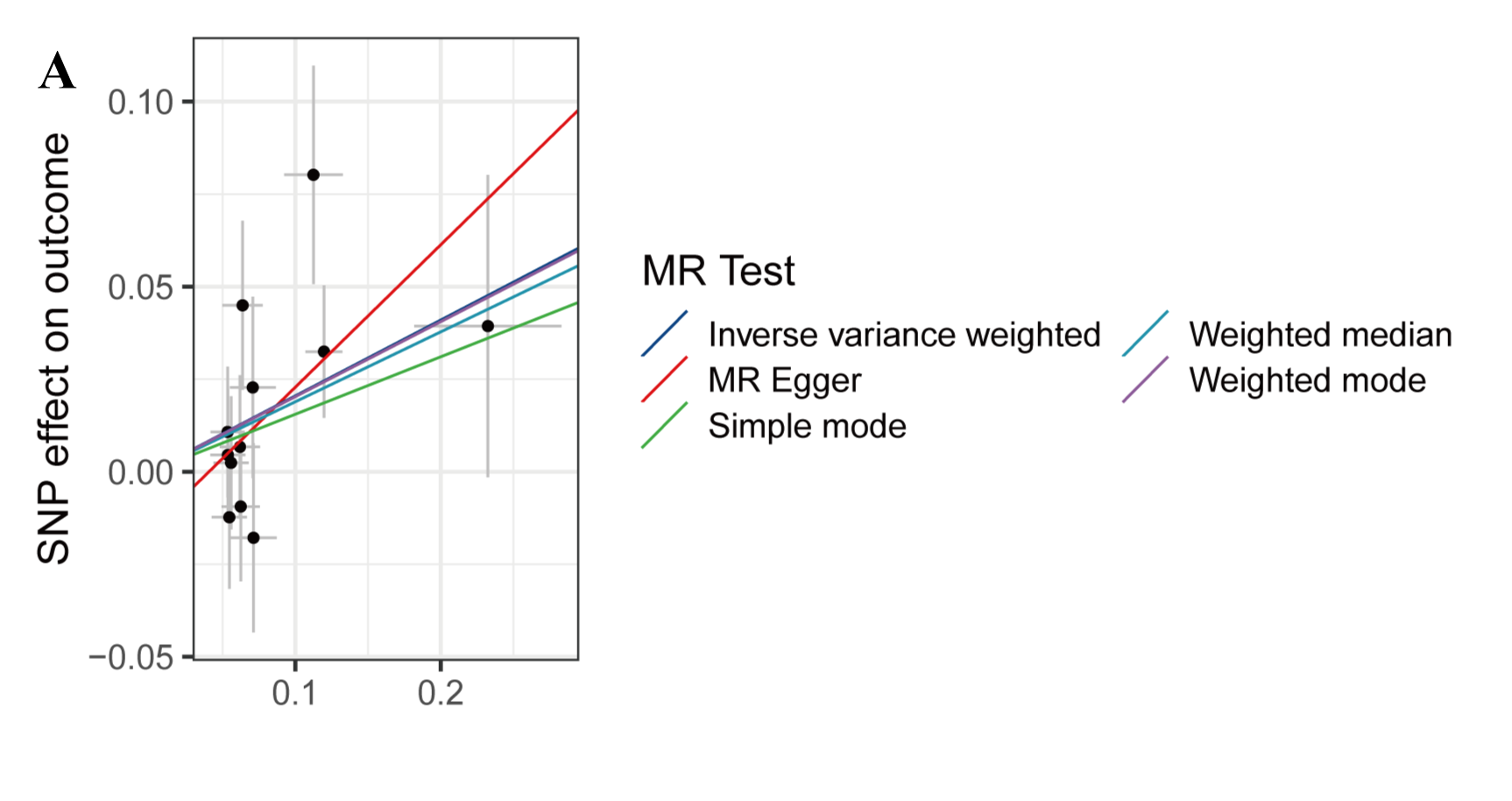


1. Analysis for " Bifidobacterium " on " Focal Epilepsy "

**Figure S6.** Scatter plots for the effect of Gut microbiota on Generalized Epilepsy.


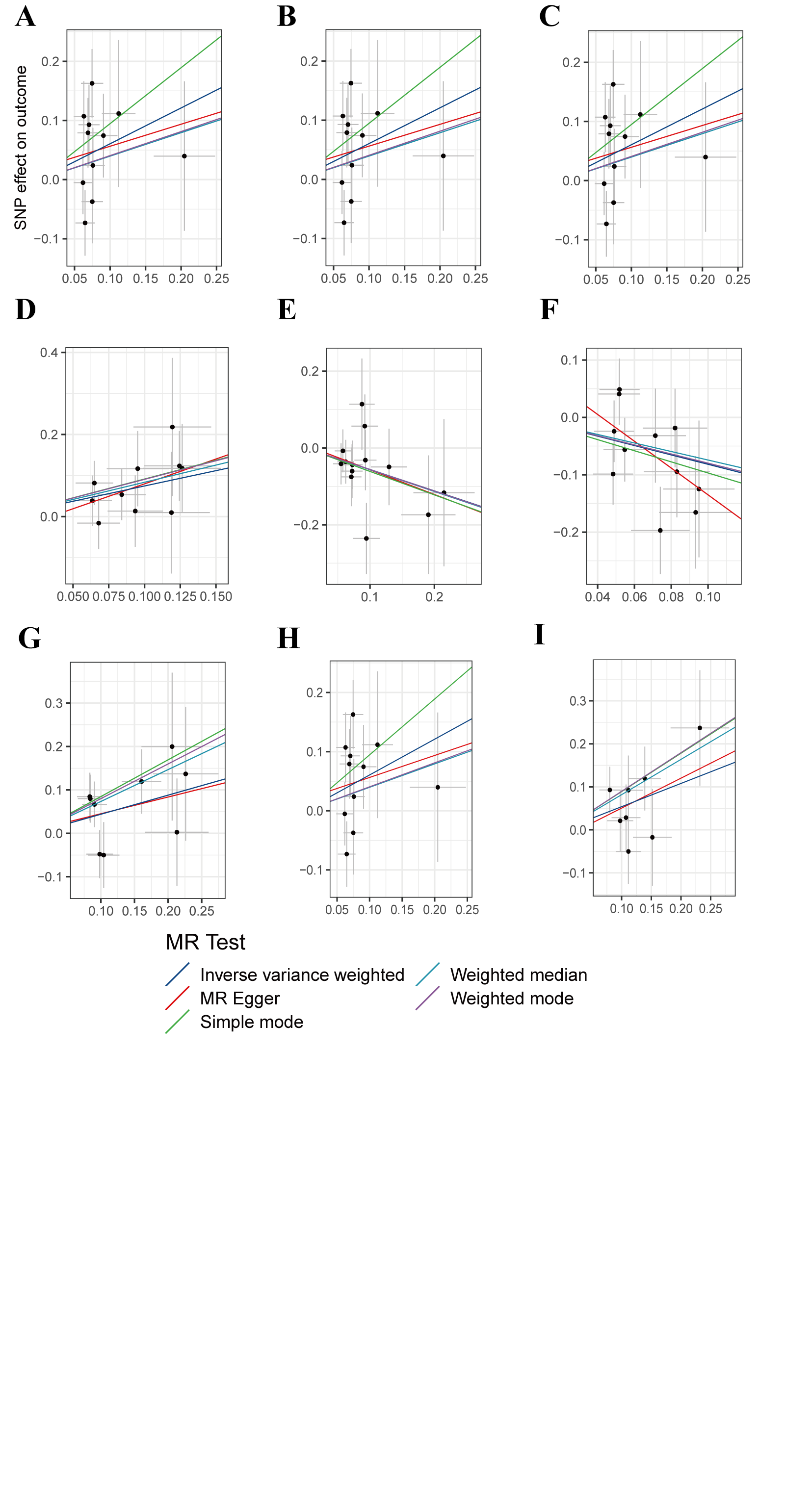


(A) Analysis for " Verrucomicrobiae " on " Generalized Epilepsy"

(B) Analysis for " Verrucomicrobiaceae " on " Generalized Epilepsy "

(C) Analysis for " Akkermansia " on " Generalized Epilepsy "

(D) Analysis for " Marvinbryantia " on " Generalized Epilepsy "

(E) Analysis for " Romboutsia " on " Generalized Epilepsy"

(F) Analysis for " Ruminiclostrium5 " on " Generalized Epilepsy "

(G) Analysis for " Gastranaerophilales " on " Generalized Epilepsy"

(H) Analysis for " Verrucomicrobiales " on " Generalized Epilepsy"

(I) Analysis for " Cyanobacteria " on " Generalized Epilepsy"

**Figure S7.** Forest plots for the effect of Gut microbiota on Epilepsy.


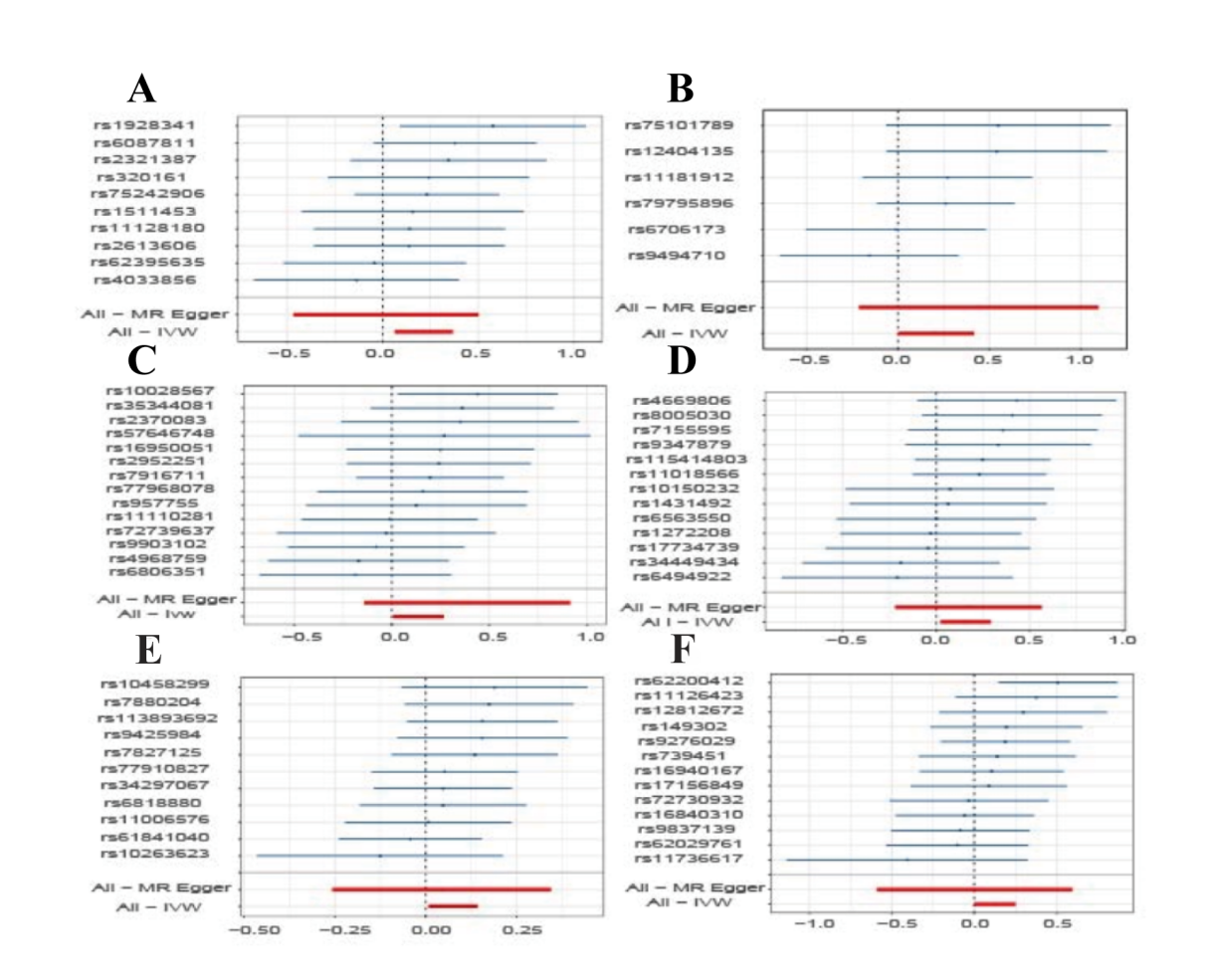


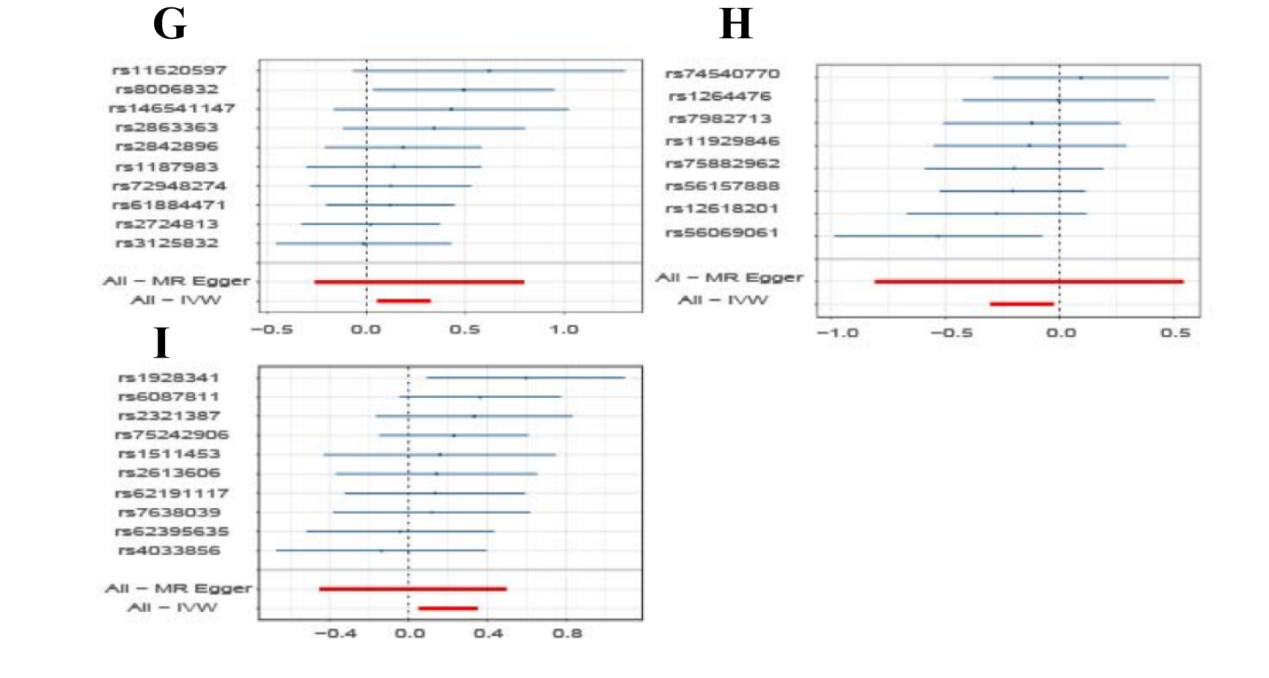


(A)Analysis for " Betaproteobacteria " on " Epilepsy "

(B) Analysis for " Gammaproteobacteria" on " Epilepsy "

(C) Analysis for " Streptococcaceae " on " Epilepsy "

(D) Analysis for " Anaerotruncus " on " Epilepsy "

(E) Analysis for " Eubacterium nodatum group " on " Epilepsy "

(F) Analysis for " Family XIII AD3011 group " on " Epilepsy "

(G) Analysis for " Marvinbryantia " on " Epilepsy "

(H) Analysis for " Phascolarctobacterium " on " Epilepsy "

(I) Analysis for " Burkholderiales " on " Epilepsy "

**Figure S8.** Forest plots for the effect of Gut microbiota on Focal Epilepsy.


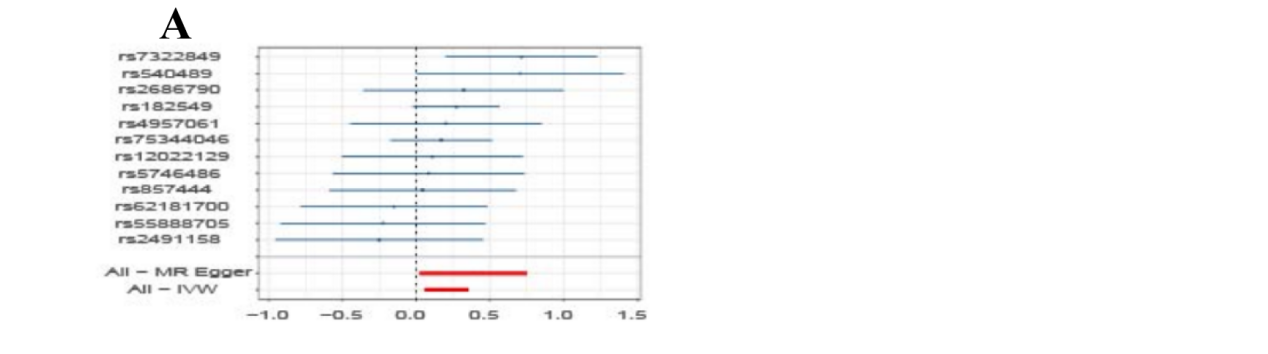


1. Analysis for " Bifidobacterium " on " Focal Epilepsy "

**Figure S9.** Forest plots for the effect of Gut microbiota on Generalized Epilepsy.


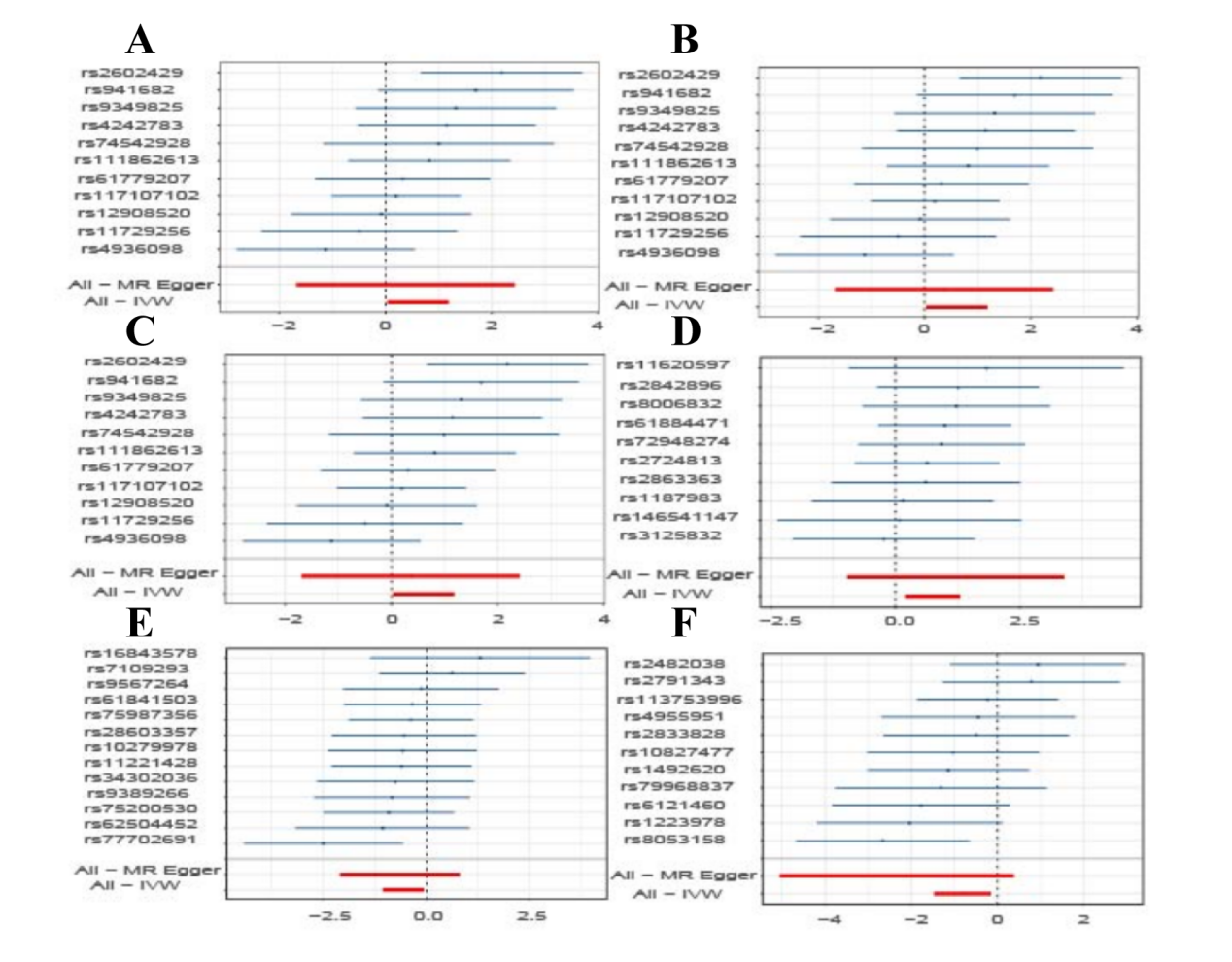


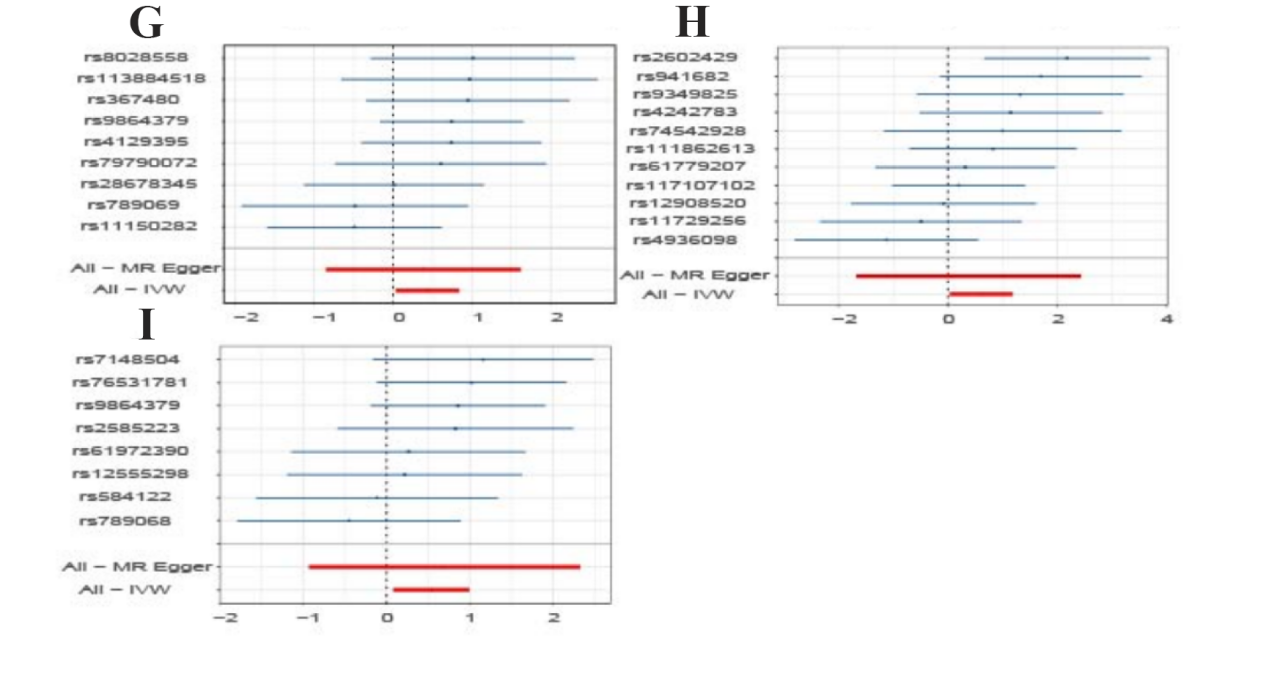


(A) Analysis for " Verrucomicrobiae " on " Generalized Epilepsy"

(B) Analysis for " Verrucomicrobiaceae " on " Generalized Epilepsy "

(C) Analysis for " Akkermansia " on " Generalized Epilepsy "

(D) Analysis for " Marvinbryantia " on " Generalized Epilepsy "

(E) Analysis for " Romboutsia " on " Generalized Epilepsy"

(F) Analysis for " Ruminiclostrium5 " on " Generalized Epilepsy "

(G) Analysis for " Gastranaerophilales " on " Generalized Epilepsy"

(H) Analysis for " Verrucomicrobiales " on " Generalized Epilepsy"

(I) Analysis for " Cyanobacteria " on " Generalized Epilepsy"

**Figure S10.** Funnel plots for the effect of Gut microbiota on Epilepsy.


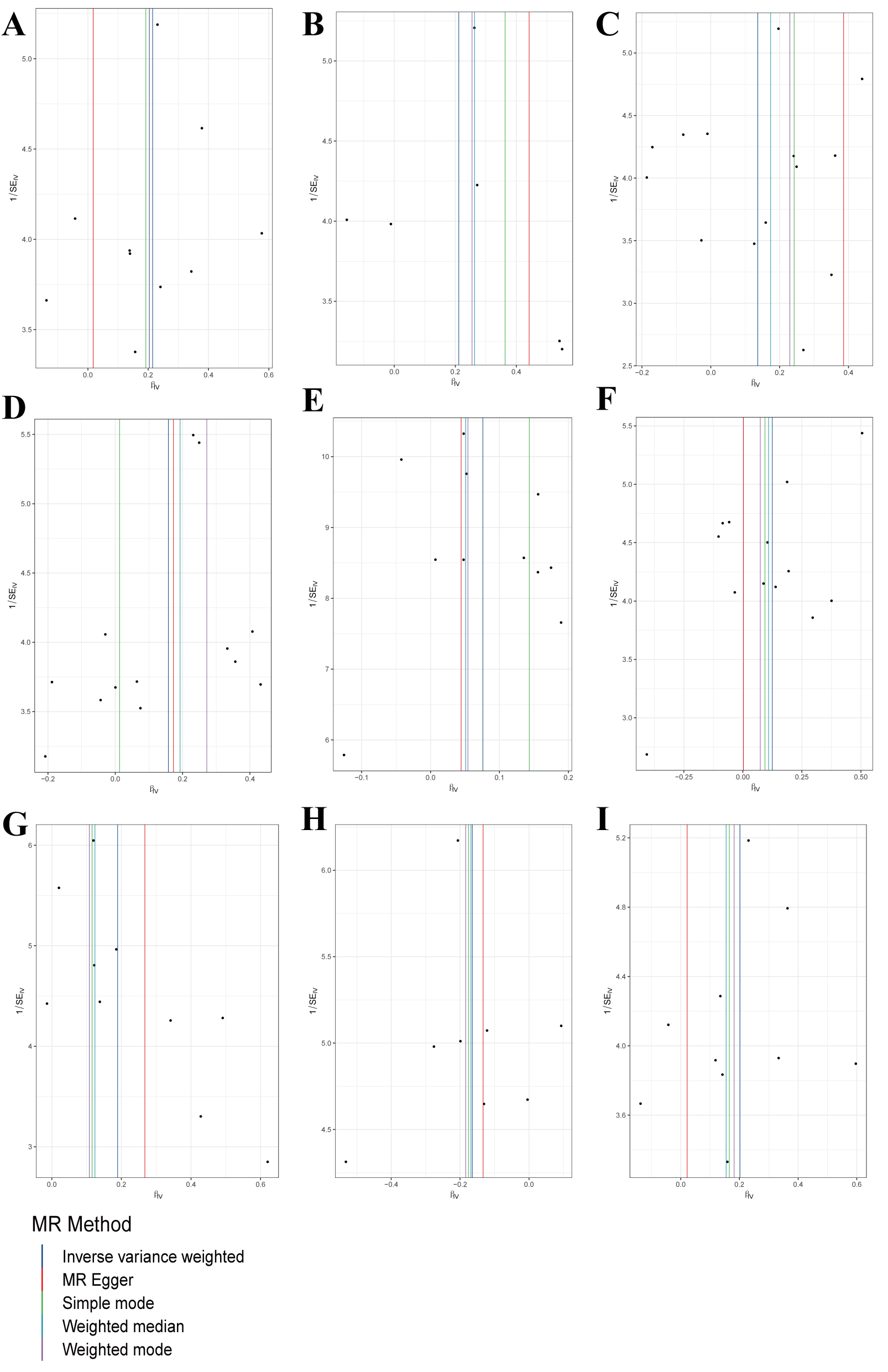


(A)Analysis for " Betaproteobacteria " on " Epilepsy "

(B) Analysis for " Gammaproteobacteria" on " Epilepsy "

(C) Analysis for " Streptococcaceae " on " Epilepsy "

(D) Analysis for " Anaerotruncus " on " Epilepsy "

(E) Analysis for " Eubacterium nodatum group " on " Epilepsy "

(F) Analysis for " Family XIII AD3011 group " on " Epilepsy "

(G) Analysis for " Marvinbryantia " on " Epilepsy "

(H) Analysis for " Phascolarctobacterium " on " Epilepsy "

(I) Analysis for " Burkholderiales " on " Epilepsy "

**Figure S11.** Funnel plots for the effect of Gut microbiota on Focal Epilepsy.


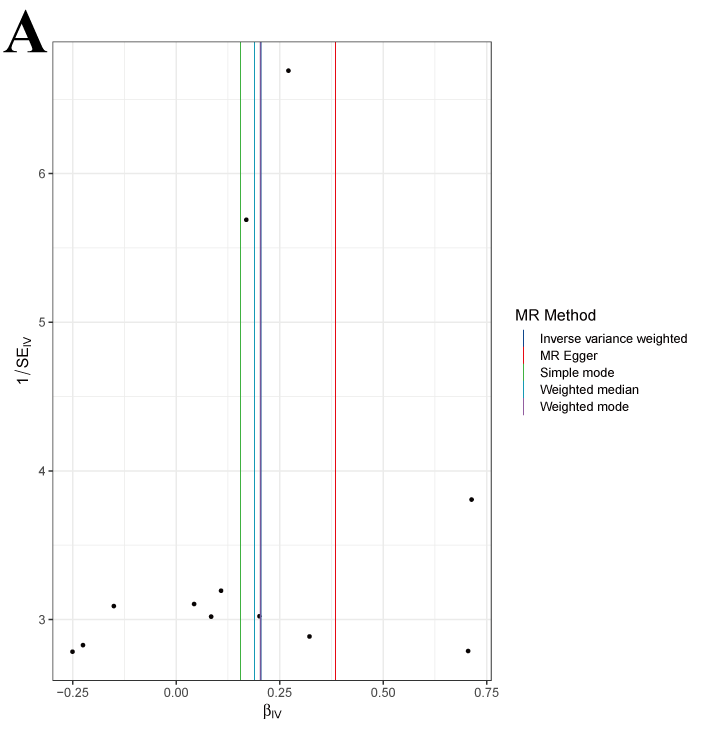


(A)Analysis for " Betaproteobacteria " on " Epilepsy "

**Figure S12.** Funnel plots for the effect of Gut microbiota on Generalized Epilepsy.


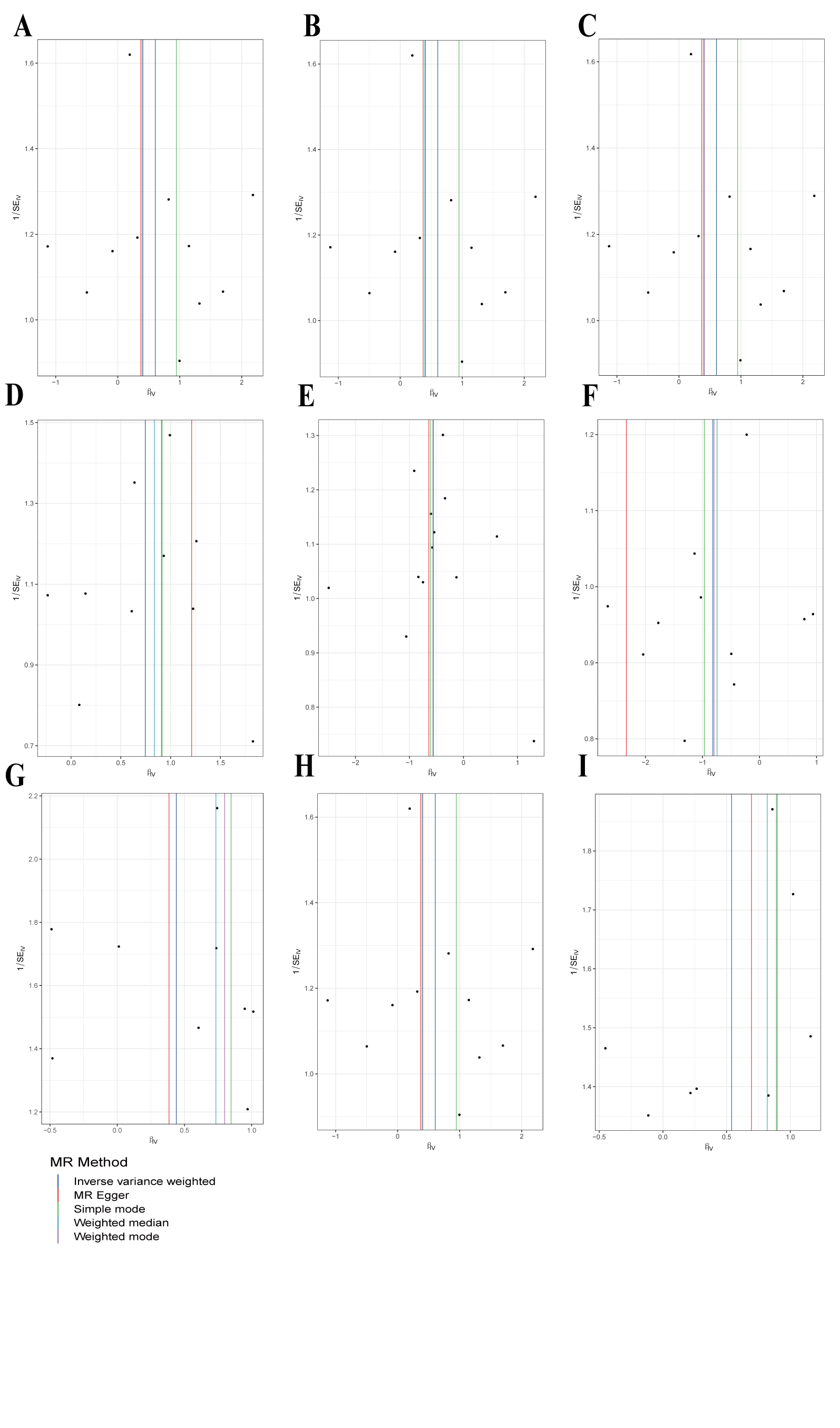


(A) Analysis for " Verrucomicrobiae " on " Generalized Epilepsy"

(B) Analysis for " Verrucomicrobiaceae " on " Generalized Epilepsy "

(C) Analysis for " Akkermansia " on " Generalized Epilepsy "

(D) Analysis for " Marvinbryantia " on " Generalized Epilepsy "

(E) Analysis for " Romboutsia " on " Generalized Epilepsy"

(F) Analysis for " Ruminiclostrium5 " on " Generalized Epilepsy "

(G) Analysis for " Gastranaerophilales " on " Generalized Epilepsy"

(H) Analysis for " Verrucomicrobiales " on " Generalized Epilepsy"

(I) Analysis for " Cyanobacteria " on " Generalized Epilepsy"

**Figure S13.** MR leave-one-out sensitivity analysis for circulating inflammatory proteins on Epilepsy.


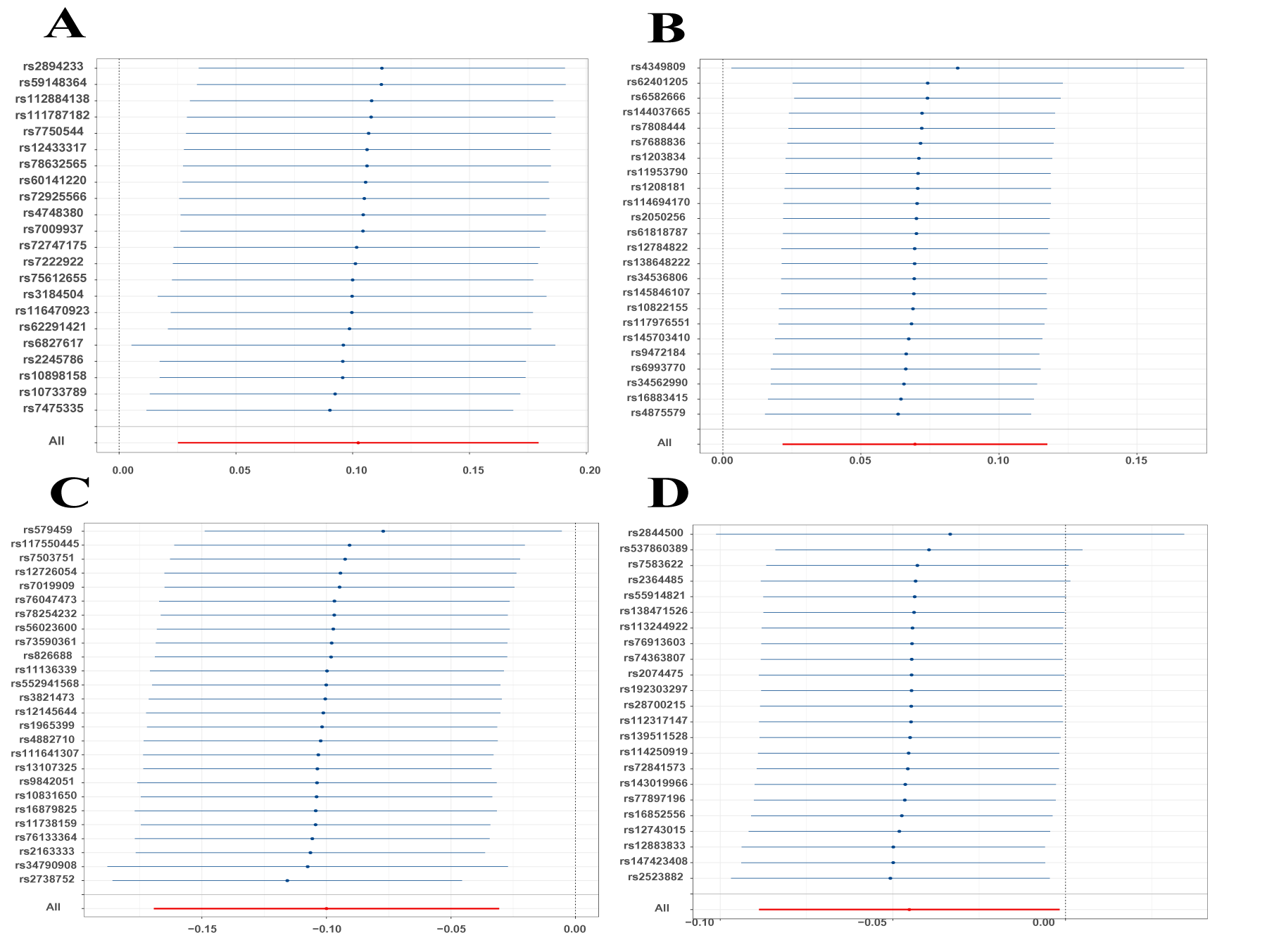


(A)Analysis for " C-X-C motif chemokine 11 levels " on " Epilepsy "

(B) Analysis for " TNF-beta levels" on " Epilepsy "

(C) Analysis for " Tumor necrosis factor ligand superfamily member 12 levels " on " Epilepsy "

(D) Analysis for " Vascular endothelial growth factor A levels " on " Epilepsy "

**Figure S14.** MR leave-one-out sensitivity analysis for circulating inflammatory proteins on Focal Epilepsy.


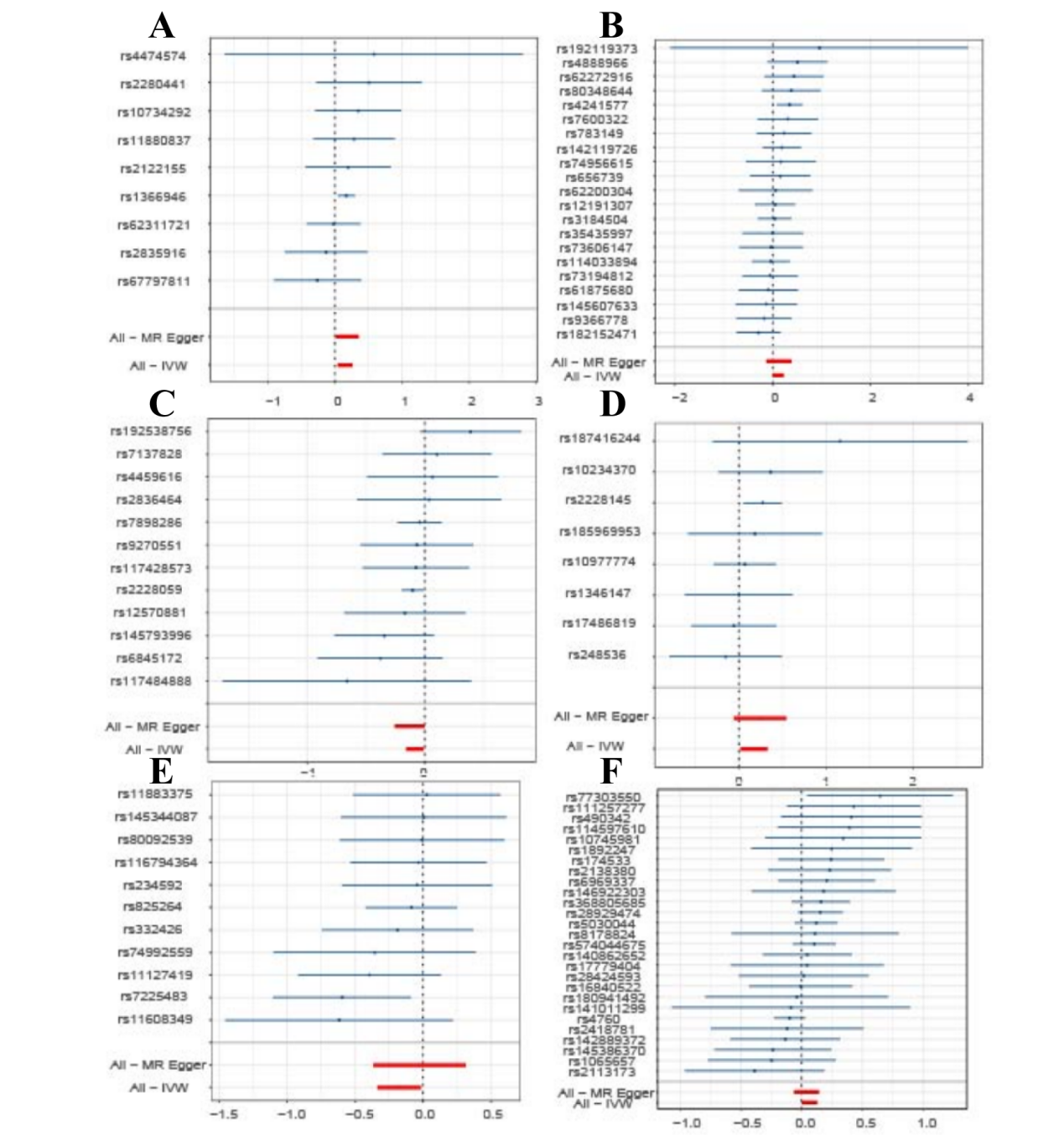


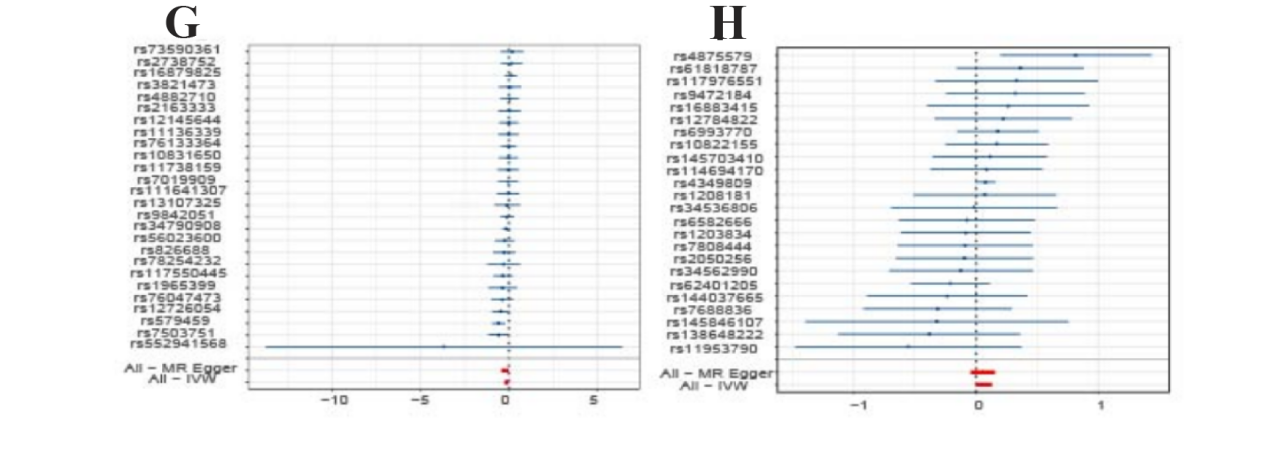


(A) Analysis for " C-X-C motif chemokine 1 levels " on " Focal Epilepsy "

(B) Analysis for " C-X-C motif chemokine 9 levels " on " Focal Epilepsy "

(C) Analysis for " Interleukin-15 receptor subunit alpha levels " on " Focal Epilepsy "

(D) Analysis for " Interleukin-6 levels " on " Focal Epilepsy "

(E) Analysis for " Leukemia inhibitory factor levels " on " Focal Epilepsy "

(F) Analysis for " TNF-related apoptosis-inducing ligand levels " on " Focal Epilepsy"

(G) Analysis for " Tumor necrosis factor ligand superfamily member 12 levels " on "Focal Epilepsy "

(H) Analysis for " Vascular endothelial growth factor A levels " on " Focal Epilepsy "

**Figure S15.** MR leave-one-out sensitivity analysis for circulating inflammatory proteins on Generalized Epilepsy.


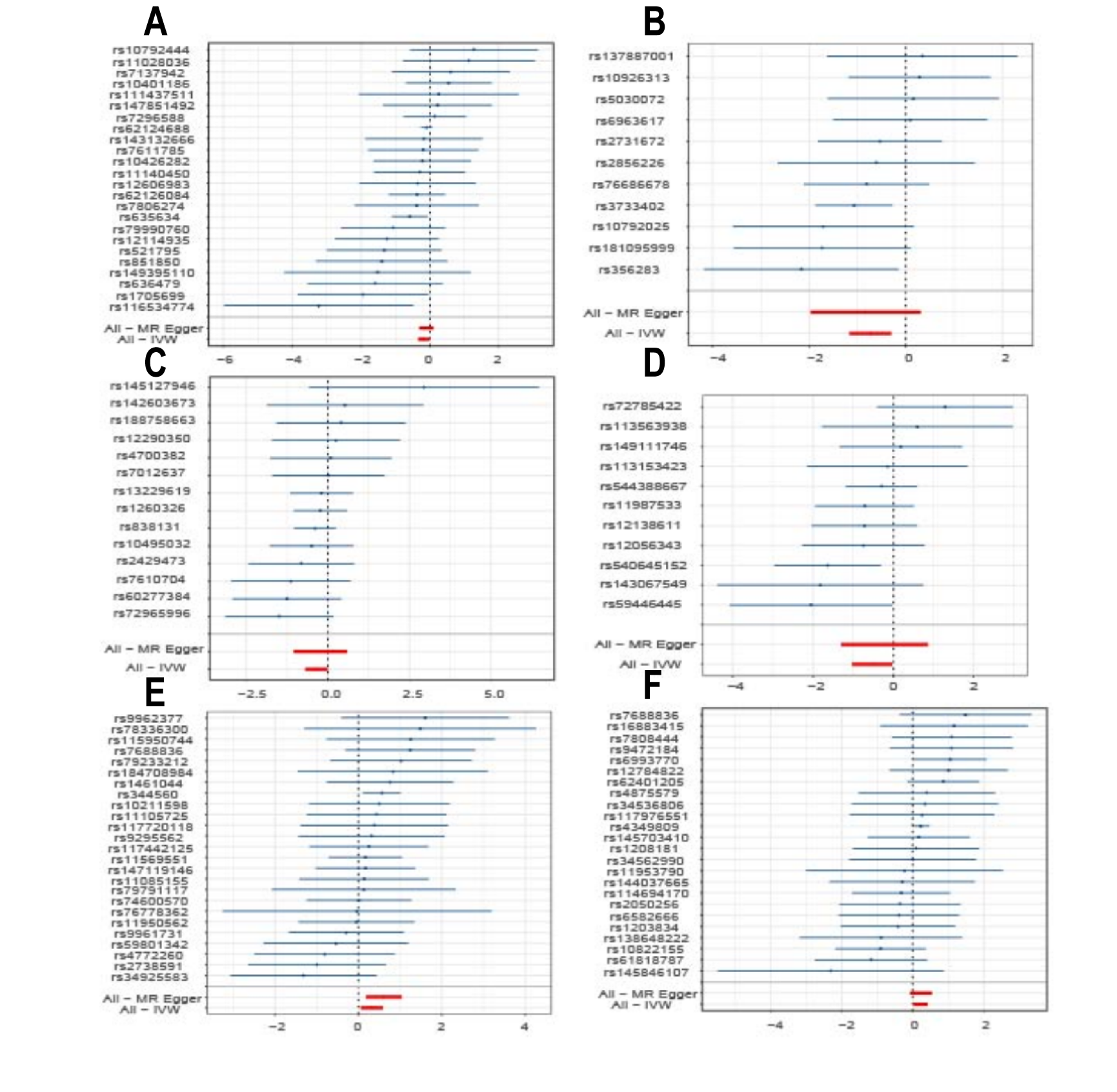


(A) Analysis for " C-C motif chemokine 25 levels " on " Generalized Epilepsy"

(B) Analysis for " Eukaryotic translation initiation factor 4E-binding protein 1 levels " on " Generalized Epilepsy "

(C) Analysis for " Fibroblast growth factor 21 levels " on " Generalized Epilepsy "

(D) Analysis for " Interleukin-20 receptor subunit alpha levels " on " Generalized Epilepsy "

(E) Analysis for " Tumor necrosis factor ligand superfamily member 14 levels " on " Generalized Epilepsy"

(F) Analysis for " Vascular endothelial growth factor A levels " on " Generalized Epilepsy "


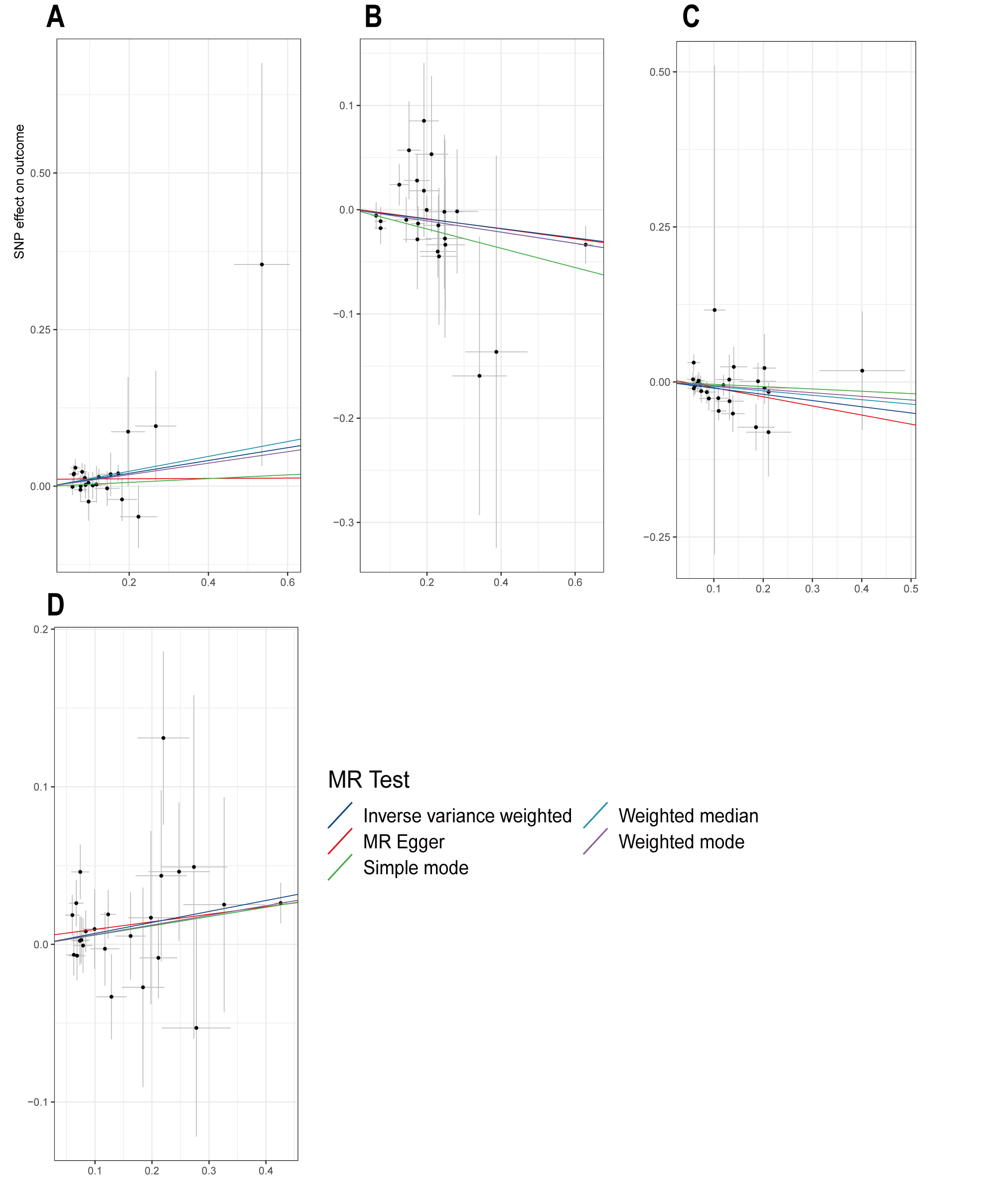
**Figure S16.** Scatter plots for the effect of circulating inflammatory proteins on Epilepsy.

(A)Analysis for " C-X-C motif chemokine 11 levels " on " Epilepsy "

(B) Analysis for " TNF-beta levels" on " Epilepsy "

(C) Analysis for " Tumor necrosis factor ligand superfamily member 12 levels " on " Epilepsy "

(D) Analysis for " Vascular endothelial growth factor A levels " on " Epilepsy "

**Figure S17.** Scatter plots for the effect of circulating inflammatory proteins on Focal Epilepsy.


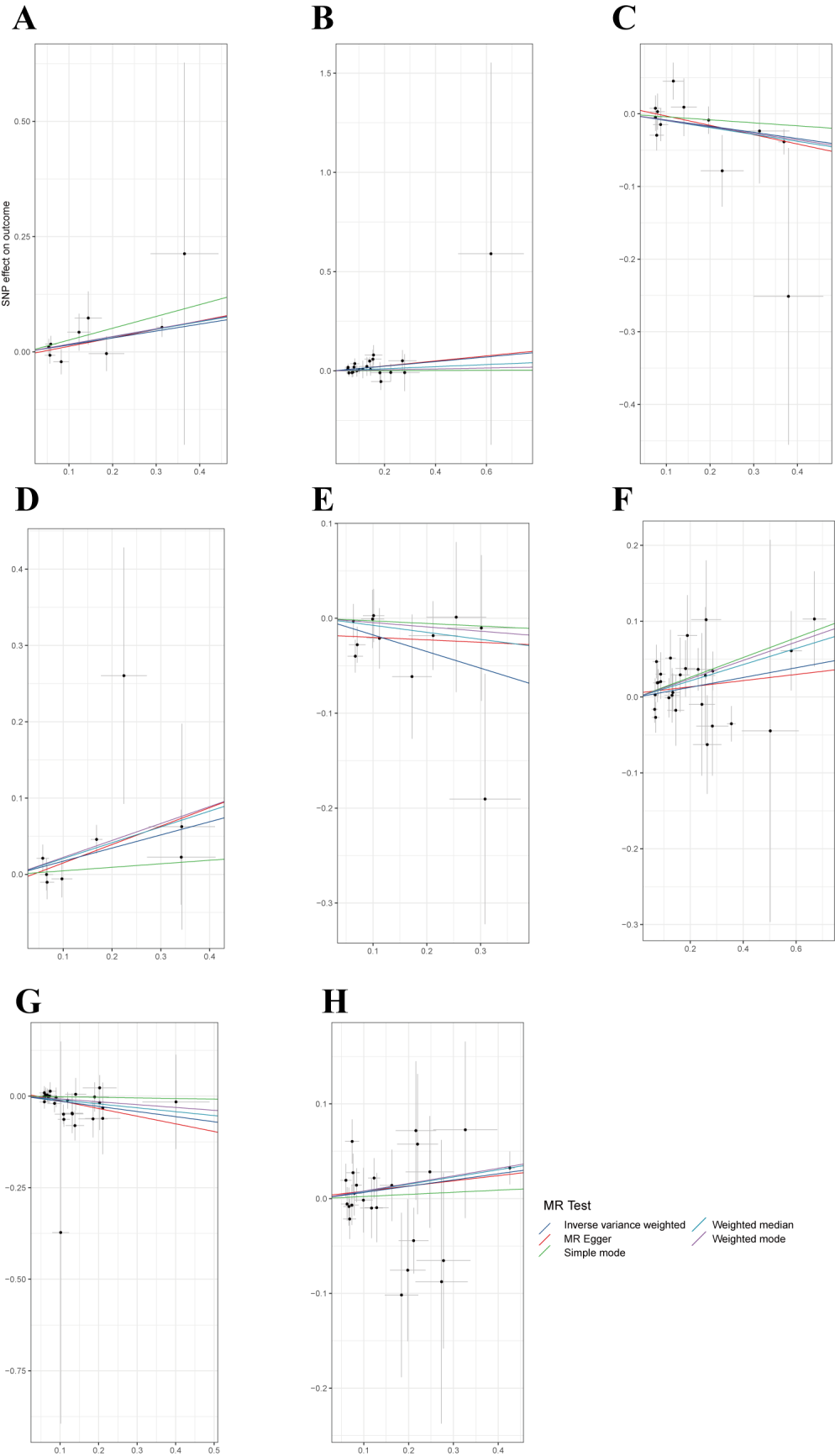


(A) Analysis for " C-X-C motif chemokine 1 levels " on " Focal Epilepsy "

(B) Analysis for " C-X-C motif chemokine 9 levels " on " Focal Epilepsy "

(C) Analysis for " Interleukin-15 receptor subunit alpha levels " on " Focal Epilepsy "

(D) Analysis for " Interleukin-6 levels " on " Focal Epilepsy "

(E) Analysis for " Leukemia inhibitory factor levels " on " Focal Epilepsy "

(F) Analysis for " TNF-related apoptosis-inducing ligand levels " on " Focal Epilepsy"

(G) Analysis for " Tumor necrosis factor ligand superfamily member 12 levels " on "Focal Epilepsy "

(H) Analysis for " Vascular endothelial growth factor A levels " on " Focal Epilepsy "

**Figure S18.** Scatter plots for the effect of circulating inflammatory proteins on Generalized Epilepsy.


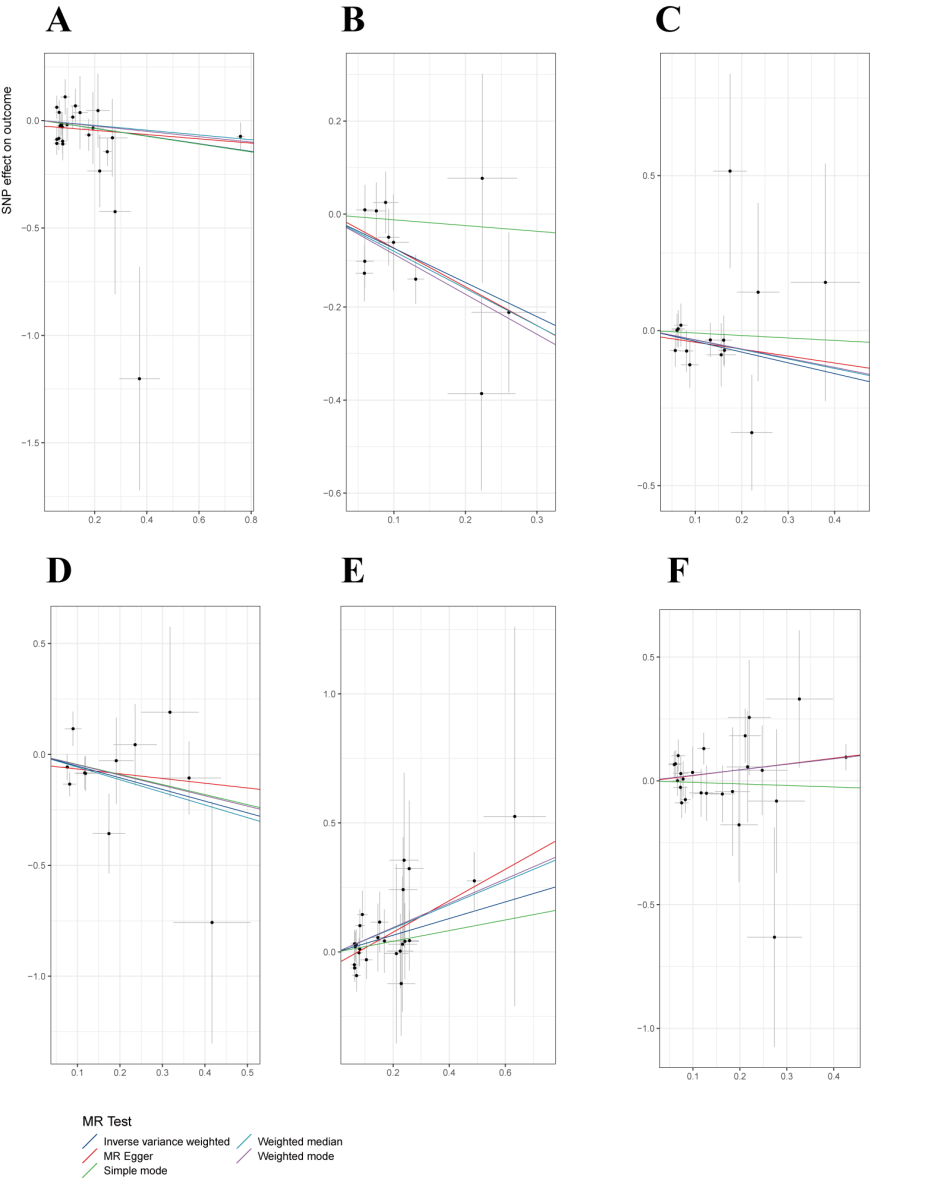


(A) Analysis for " C-C motif chemokine 25 levels " on " Generalized Epilepsy"

(B) Analysis for " Eukaryotic translation initiation factor 4E-binding protein 1 levels " on " Generalized Epilepsy "

(C) Analysis for " Fibroblast growth factor 21 levels " on " Generalized Epilepsy "

(D) Analysis for " Interleukin-20 receptor subunit alpha levels " on " Generalized Epilepsy "

(E) Analysis for " Tumor necrosis factor ligand superfamily member 14 levels " on " Generalized Epilepsy"

(F) Analysis for " Vascular endothelial growth factor A levels " on " Generalized Epilepsy "

**Figure S19.** Forest plots for the effect of circulating inflammatory proteins on Epilepsy.


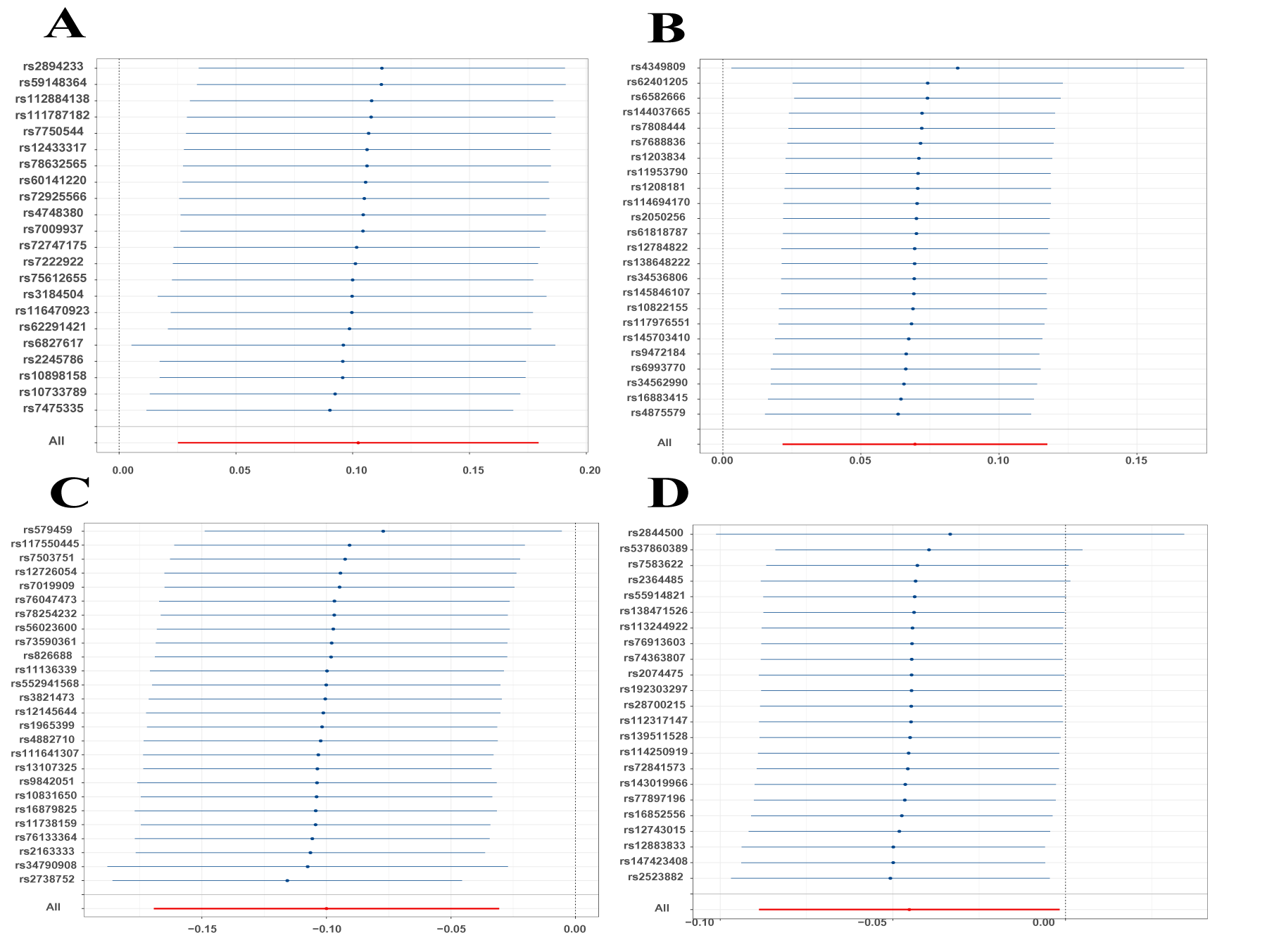


(A)Analysis for " C-X-C motif chemokine 11 levels " on " Epilepsy "

(B) Analysis for " TNF-beta levels" on " Epilepsy "

(C) Analysis for " Tumor necrosis factor ligand superfamily member 12 levels " on " Epilepsy "

(D) Analysis for " Vascular endothelial growth factor A levels " on " Epilepsy "

**Figure S20.** Forest plots for the effect of circulating inflammatory proteins on Focal Epilepsy.


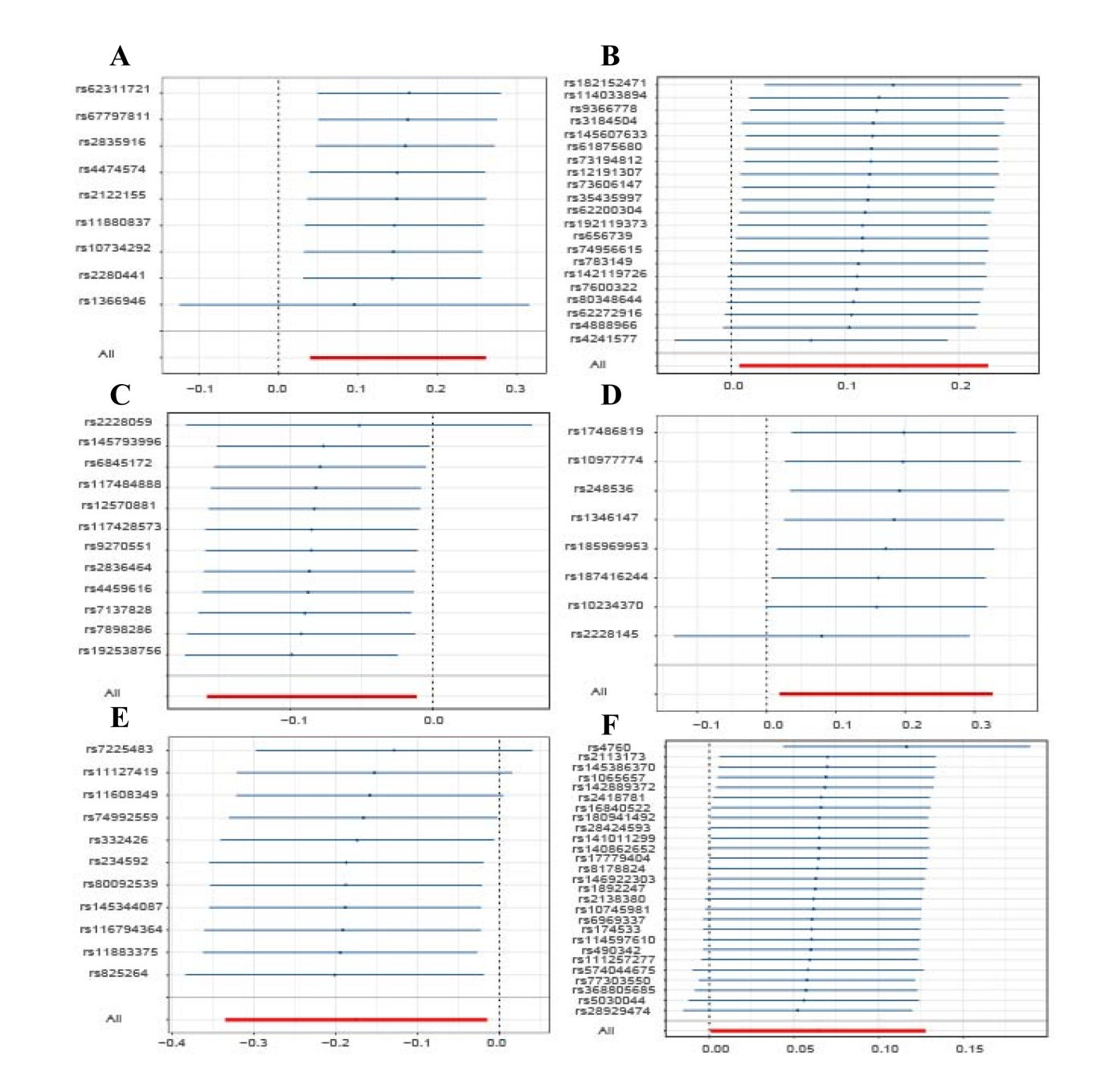


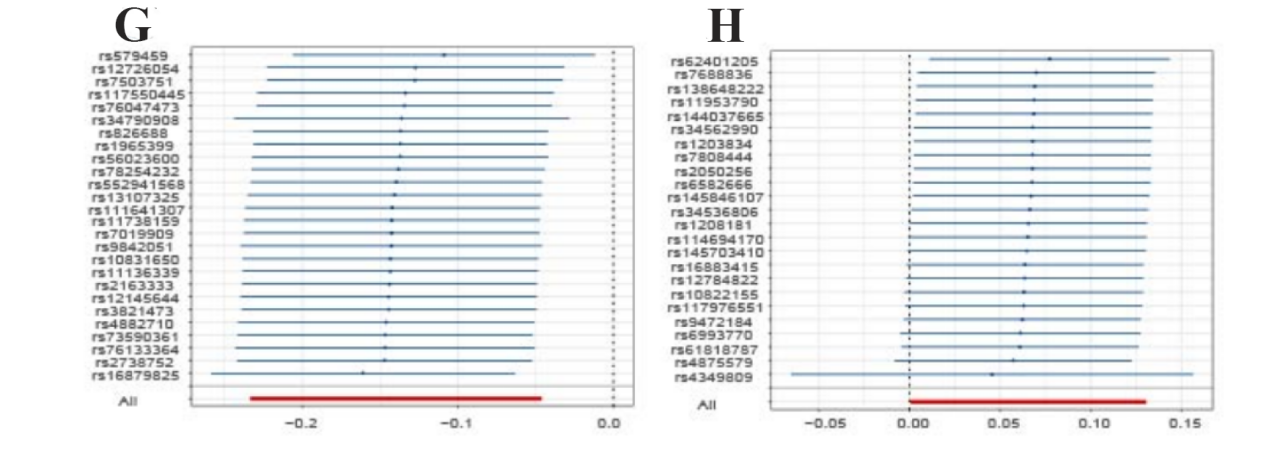


(A) Analysis for " C-X-C motif chemokine 1 levels " on " Focal Epilepsy "

(B) Analysis for " C-X-C motif chemokine 9 levels " on " Focal Epilepsy "

(C) Analysis for " Interleukin-15 receptor subunit alpha levels " on " Focal Epilepsy "

(D) Analysis for " Interleukin-6 levels " on " Focal Epilepsy "

(E) Analysis for " Leukemia inhibitory factor levels " on " Focal Epilepsy "

(F) Analysis for " TNF-related apoptosis-inducing ligand levels " on " Focal Epilepsy"

(G) Analysis for " Tumor necrosis factor ligand superfamily member 12 levels " on "Focal Epilepsy "

(H) Analysis for " Vascular endothelial growth factor A levels " on " Focal Epilepsy "

**Figure S21.** Forest plots for the effect of circulating inflammatory proteins on Generalized Epilepsy.


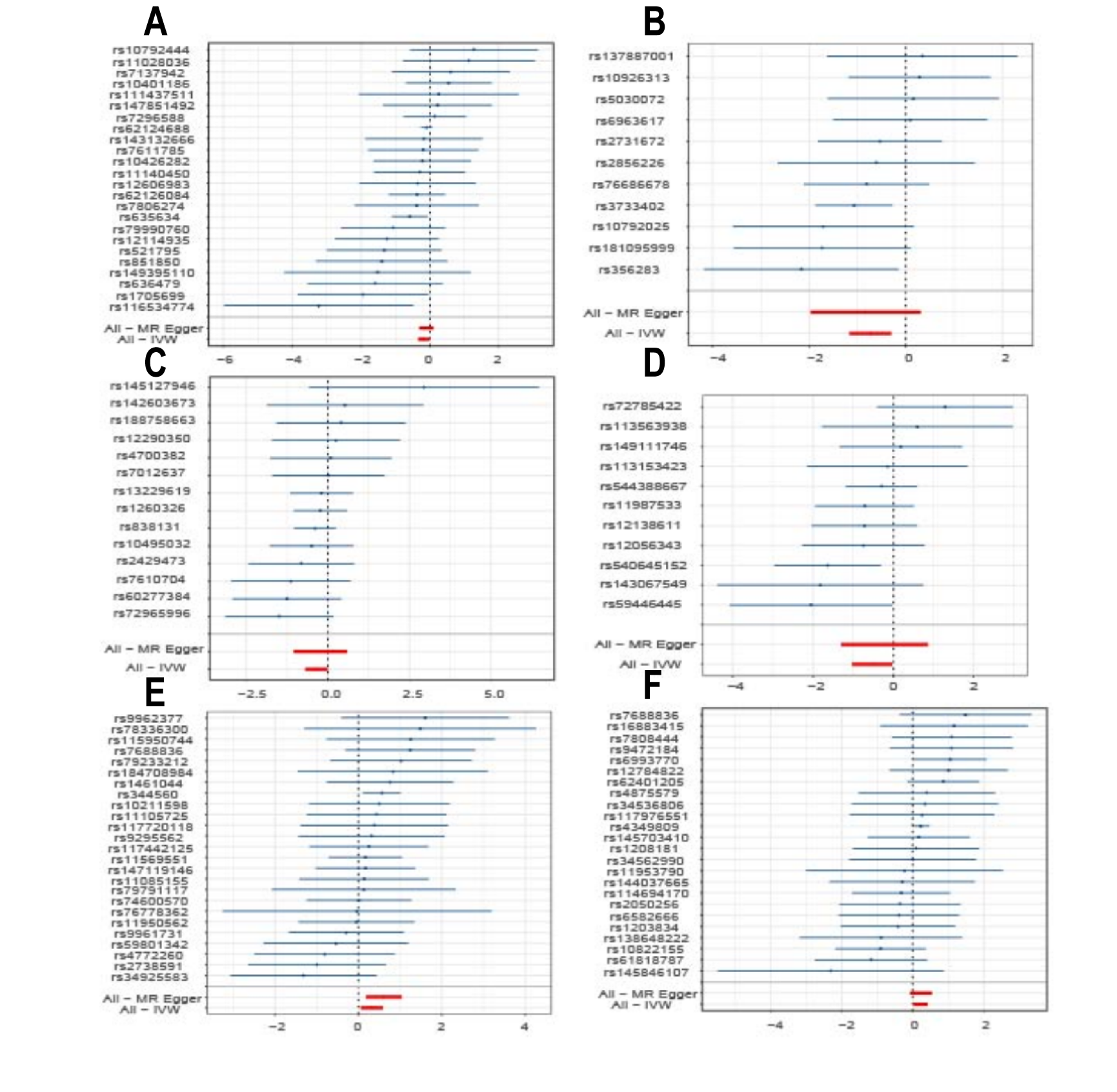


(A) Analysis for " C-C motif chemokine 25 levels " on " Generalized Epilepsy"

(B) Analysis for " Eukaryotic translation initiation factor 4E-binding protein 1 levels " on " Generalized Epilepsy "

(C) Analysis for " Fibroblast growth factor 21 levels " on " Generalized Epilepsy "

(D) Analysis for " Interleukin-20 receptor subunit alpha levels " on " Generalized Epilepsy "

(E) Analysis for " Tumor necrosis factor ligand superfamily member 14 levels " on " Generalized Epilepsy"

(F) Analysis for " Vascular endothelial growth factor A levels " on " Generalized Epilepsy

**Figure S22.** Funnel plots for the effect of circulating inflammatory proteins on Epilepsy.


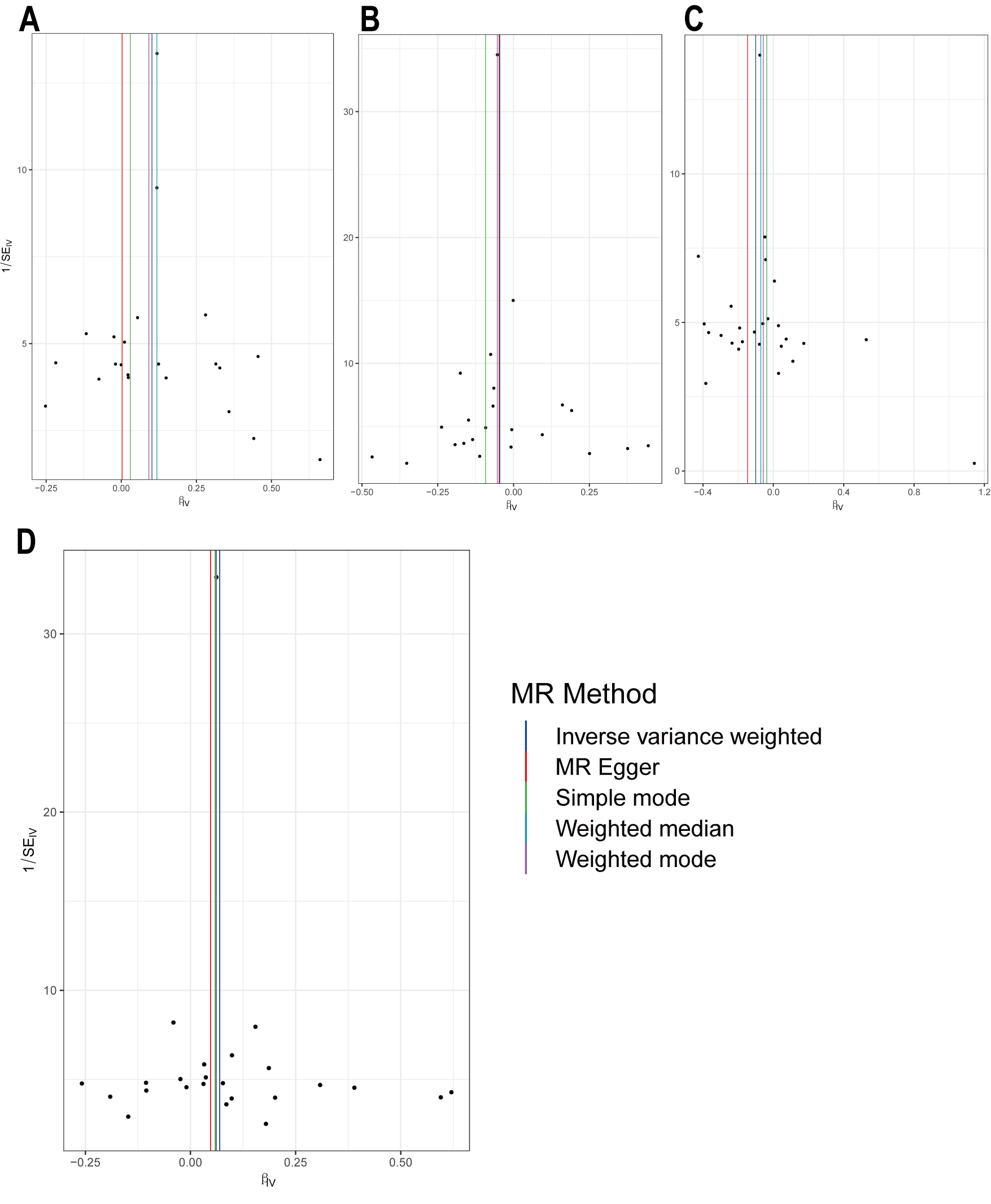


(A)Analysis for " C-X-C motif chemokine 11 levels " on " Epilepsy "

(B) Analysis for " TNF-beta levels" on " Epilepsy "

(C) Analysis for " Tumor necrosis factor ligand superfamily member 12 levels " on " Epilepsy "

(D) Analysis for " Vascular endothelial growth factor A levels " on " Epilepsy "

**Figure S23.** Funnel plots for the effect of circulating inflammatory proteins on Focal Epilepsy.


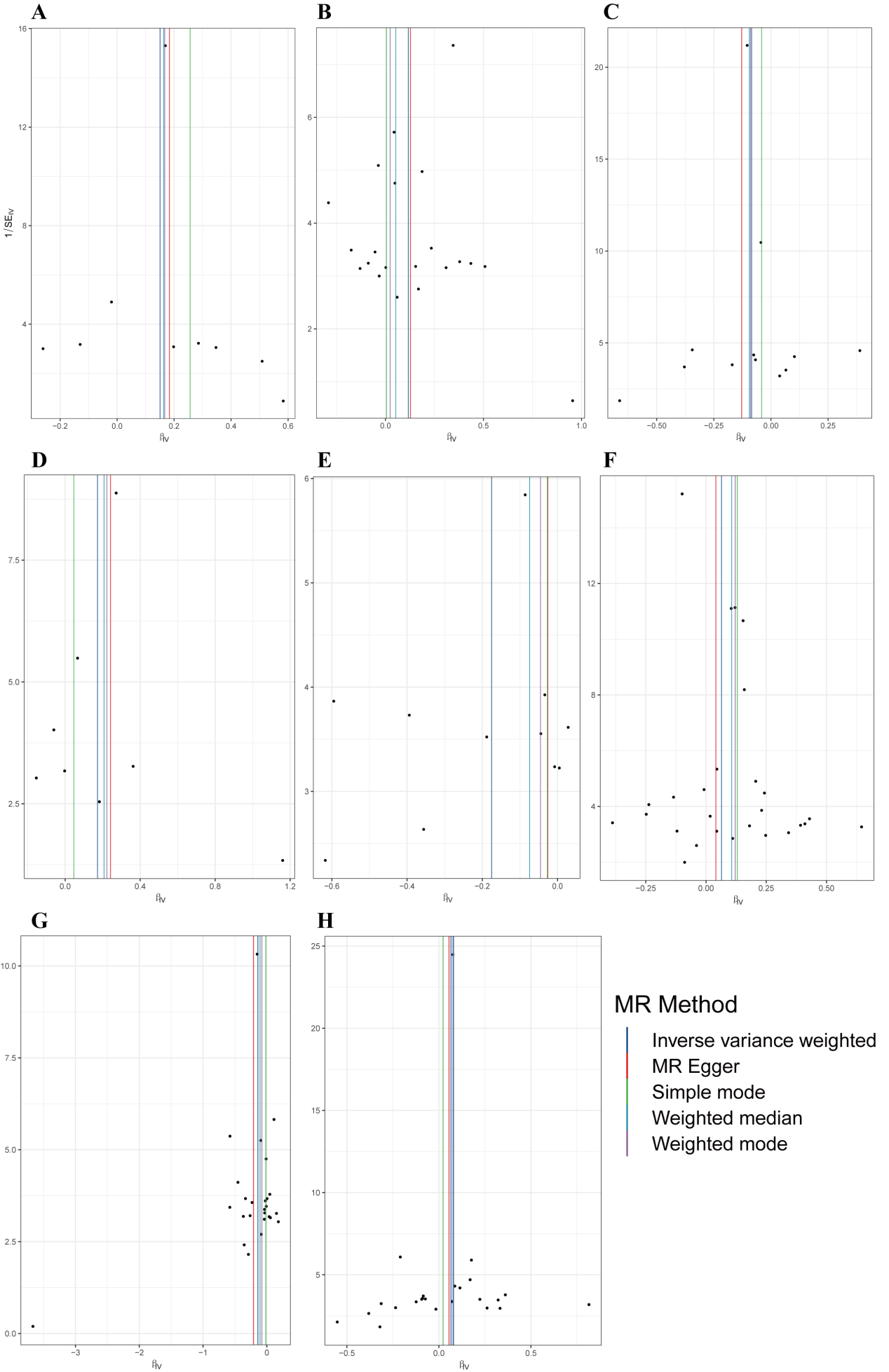


(A) Analysis for " C-X-C motif chemokine 1 levels " on " Focal Epilepsy "

(B) Analysis for " C-X-C motif chemokine 9 levels " on " Focal Epilepsy "

(C) Analysis for " Interleukin-15 receptor subunit alpha levels " on " Focal Epilepsy "

(D) Analysis for " Interleukin-6 levels " on " Focal Epilepsy "

(E) Analysis for " Leukemia inhibitory factor levels " on " Focal Epilepsy "

(F) Analysis for " TNF-related apoptosis-inducing ligand levels " on " Focal Epilepsy"

(G) Analysis for " Tumor necrosis factor ligand superfamily member 12 levels " on "Focal Epilepsy "

(H) Analysis for " Vascular endothelial growth factor A levels " on " Focal Epilepsy "

**Figure S24.** Funnel plots for the effect of circulating inflammatory proteins on Generalized Epilepsy.


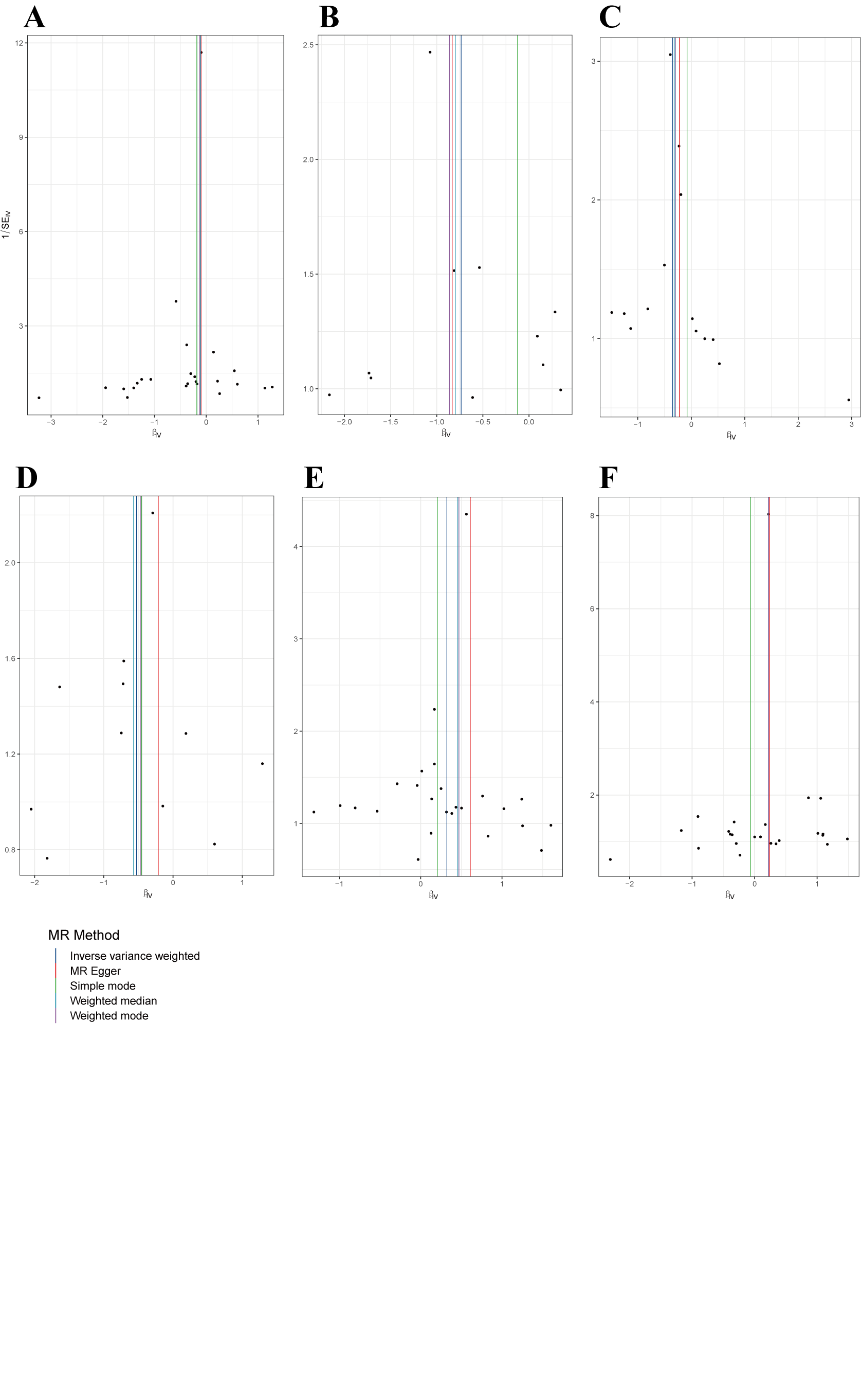


(A) Analysis for " C-C motif chemokine 25 levels " on " Generalized Epilepsy"

(B) Analysis for " Eukaryotic translation initiation factor 4E-binding protein 1 levels " on " Generalized Epilepsy "

(C) Analysis for " Fibroblast growth factor 21 levels " on " Generalized Epilepsy "

(D) Analysis for " Interleukin-20 receptor subunit alpha levels " on " Generalized Epilepsy "

(E) Analysis for " Tumor necrosis factor ligand superfamily member 14 levels " on " Generalized Epilepsy"

(F) Analysis for " Vascular endothelial growth factor A levels " on " Generalized Epilepsy
